# Unveiling peripheral symmetric acceptors coupling with tetrathienylbenzene core to promote electron transfer dynamics in organic photovoltaics

**DOI:** 10.1038/s41598-024-71777-6

**Published:** 2024-09-11

**Authors:** Muhammad Khalid, Aiman Jabbar, Shahzad Murtaza, Muhammad Arshad, Ataualpa A. C. Braga, Tansir Ahamad

**Affiliations:** 1 Institute of Chemistry, Khwaja Fareed University of Engineering & Information Technology, Rahim Yar Khan, 64200 Pakistan; 2 Centre for Theoretical and Computational Research, Khwaja Fareed University of Engineering & Information Technology, Rahim Yar Khan, 64200 Pakistan; 3 Industry Solutions, Northern Alberta Institute of Technology, Edmonton, AB Canada; 4 Departamento de Química Fundamental, Instituto de Química, Universidade de São Paulo, Av. Prof. Lineu Prestes, 748, São Paulo, 05508-000 Brazil; 5 Department of Chemistry, College of Science, King Saud University, Riyadh 11451, Saudi Arabia

**Keywords:** Thiophene, A**–**π**–**A, Charge transfer, DFT, Photovoltaic properties, Chemistry, Materials science

## Abstract

Non-fullerene organic compounds are promising materials for advanced photovoltaic devices. The photovoltaic and electronic properties of the derivatives (**TTBR** and **TTB1-TTB6**) were determined by employing density functional theory (DFT) and time-dependent density functional theory (TD-DFT) analyses using the M06/6-311G(d,p) functional. To enhance the effectiveness of fullerene-free organic photovoltaic cells, modifications were applied to end-capped acceptors by using strong electron-withdrawing moieties. The structural tailoring showed a significant electronic impact for HOMO and LUMO for all chromophores, resulting in decreased band gaps (3.184–2.540 eV). Interestingly, all the designed derivatives exhibited broader absorption spectra in the range of 486.365–605.895 nm in dichloromethane solvent*.* Among all derivatives, **TTB5** was observed to be the promising candidate because of its lowest energy gap (2.54 eV) and binding energy (0.494 eV) values, along with the bathochromic shift (605.895 nm). These chromophores having an A**–**π**–**A framework might be considered promising materials for efficient organic cells.

## Introduction

Nowadays, many organic-based materials are designed because of their remarkable characteristics, which include lightweight, transparency, mobility and high efficiency for the photovoltaic solar cells^1^. Organic photovoltaics (OPVs) have significant potential in addressing the world's upcoming energy demands^2^. NFAs-based solar cells are considered as highly promising photovoltaic materials, in terms of cost-effectiveness, enhanced power conversion efficiency (PCE) and thermal stability^3^. They significantly increase the charge carrier mobility within a molecule due to the high intramolecular charge transfer (ICT) rate^4^. Recent research studies show that NFAs have increased the PCEs of OSCs from 16.2 to 18.2%^5^. They are usually categorized into two types: polymer solar cells (PSCs) and small molecular acceptors (SMAs) solar cells. Organic solar cells (OSCs) using polymer acceptors have achieved a PCE above 10%^6^. The SMAs solar cells offer benefits as compared to polymer solar cells (PSCs) due to their efficient molecular structure and extra purity^7^.

These SMAs have lower exciton binding energies, resulting in enhanced charge separation and transport efficiency. Furthermore, their energy level alignment with donor materials is more advantageous, enhancing photovoltaic properties. In addition, these SMAs have the potential to offer improved stability and enhance suitability for high-efficiency OSCs as compared to other acceptors^8^. Utilizing the electron-withdrawing groups as acceptor moieties at terminals along with a core containing electron-donating units can effectively decrease band gaps, increase binding energies, broaden absorption spectra, enhance exciton dissociation rate and open-circuit voltage in the organic solar cells (OSCs)^9^.

Various NFAs molecules consisting of conjugated polymers and electron-withdrawing moieties with molecular configurations such as A–π–D–π–A, D–π–D–π–A, D–π–A, A–π–A–π–A and A–π–A have been reported^10^. These frameworks increase the range of wavelengths of absorption, reduce the energy difference between the HOMO and LUMO and improve the localization of electrons, leading to favorable photovoltaic performance^11^. In addition, various morphologies can have an impact on the dipole moments and transition probabilities, which in turn affect the intensity and shape of the absorption peaks. Hence, chromophores' structural qualities substantially influence their electronic attributes and optical absorption patterns, manifesting as alterations in the energies of molecular orbitals and the spectral properties observed in UV–Vis analysis.

Linear-shaped OSCs based materials utilizing an acceptor–π–acceptor (A–π–A) backbone are successfully designed, where an electron-rich core is attached with effective electron-withdrawing units^12^. Increasing the core size causes a red-shift in absorption spectra, increases energy levels and improves electron mobility^13^. Such NFAs solar cells are advantageous due to their ability to efficiently capture a significant quantity of sunlight, which directly boosts the photocurrent in these photovoltaic devices^14^.

Further, the π-conjugated cores have gained significant interest in the OSCs because of their enhanced PCEs and fill factor (FF) values^15^. Compounds having a π-linker thiophene are known for effective electro-thermal, optical and photovoltaic characteristics. This is owing to the increased conjugation within the molecular framework and electron-donating effect of the sulfur atom in charge distribution. Thiophene acts as donor as well as π-spacer in molecular structure to improve photovoltaic characteristics^16^.

Thiophene-based cores have attracted considerable interest in OSCs due to their high resonance, lower electrophilic reactivity, improved charge transfer mobility and planar structure as compared to benzene^17^. The tetra thienyl benzene (TTB) core has an extended π-conjugated system due to its alternate single and double bonds. This conjugation allows the molecule to absorb a broader spectrum of light wavelengths, including those in the near-infrared (NIR) region and visible region^18^. Considering the importance of TTB core^19^, **TTBR** and **TTB1-TTB6** chromophores are modified by introducing strong electron-donating group in center which acts as a π-linker and electron-accepting end-capped groups at terminals. According to literature, the structural tailoring at the terminal ends improves the photovoltaic characteristics of the designed chromophores and facilitates efficient charge transport^20^.

DFT and TD-DFT analyses of designed derivatives (**TTBR** and **TTB1-TTB6**) are performed in this study. Following this, various parameters such as frontier molecular orbitals (FMOs), density of states (DOS), global reactivity parameters (GRPs), UV–Visible absorption, transition density matrix (TDM) and open circuit voltage (*V*_oc_) are analyzed to investigate the photovoltaic response^21^. The designed chromophores showed favorable photovoltaic characteristics and power conversion efficiencies as a result of significant charge transfer process. Thus, they are good candidates to provide electricity at a lower cost than the first and second-generation solar cell technologies in near future.

### Computational procedure

The chromophores (**TTBR** and **TTB1-TTB6**) were subjected to theoretical calculations using the Gaussian 09 software^22^. To perform the DFT\TD-DFT analyses, their structures were optimized at the Minnesota 06 exchange-correlational functional (M06)^23^ and 6-311G(d,p) basis set^24^. The optoelectronic and photovoltaic properties which included UV–Visible, FMOs, DOS, *V*_oc_, electron–hole and TDM analyses were determined for the studied compounds at the afore-mentioned functional. Moreover, the HOMO–LUMO band gaps were utilized to estimate their GRPs. TD-DFT analysis was used to analyze the excited state property such as UV–Visible spectra and FMOs. The absorption spectra of studied chromophores were predicted in dichloromethane solvent. To interpret the data from the input files, software programs such as Avogadro^25^, Origin Pro 8.5^26^, Chemcraft^27^, GausSum^28^, Multiwfn 3.7^29^, Gauss View 6.0^30^ and PyMOlyze 2.0^31^ were utilized.

## Results and discussion

**Fig. 1:**
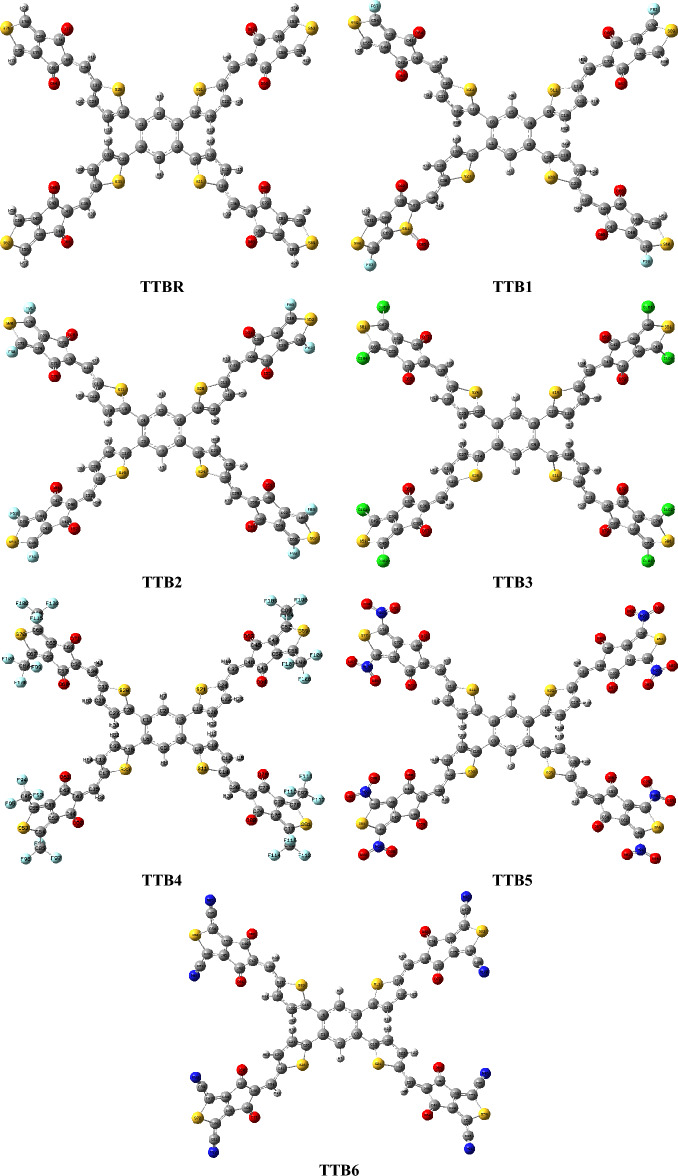
Optimized structures of the titled compounds (**TTBR** and **TTB1-TTB6**).

The selection of π–conjugated core is an essential phenomenon that significantly influences the photovoltaic characteristics of the A–π–A configured organic compounds^32^. Moreover, the literature study highlights the significance of structural modifications for enhancing optoelectronic properties^33^. The objective of current study is to design advanced organic materials based on the TTB unit accompanied by the small molecular acceptors (SMAs) to anticipate their optoelectronic and photovoltaic properties for potential use in the organic solar cells (OSCs). Figure S1 shows the structural modeling of **TTB** parent into **TTBR**. This reference compound is further modified via the alteration with the end-capped acceptor moieties such as 5-methylene-4-*H*-cyclopenta[c]thiophene-4,6(5-*H*)-dione (A1) in **TTBR**, 1-fluoro-5-methylene-4-*H*-cyclopenta[c]thiophene-4,6(5-*H*)-dione (A2) in **TTB1**, 1,3-difluoro-5-methylene-4-*H*-cyclopenta[c]thiophene-4,6(5-*H*)-dione (A3) in **TTB2**, 1,3-dichloro-5-methylene-4-*H*-cyclopenta[c]thiophene-4,6(5-*H*)-dione (A4) in **TTB3**, 5-methylene-1,3-bis(trifluoromethyl)-4-*H*-cyclopenta[c]thiophene-4,6(5-*H*)-dione (A5) in **TTB4**, 5-methylene-1,3-dinitro-4-*H*-cyclopenta[c]thiophene-4,6(5-*H*)-dione (A6) in **TTB5** and 5-methylene-4,6-dioxo-5,6-dihydro-4-*H*-cyclopenta[c]thiophene-1,3-dicarbonitrile (A7) in **TTB6**. Their structures are represented in the Fig. S2. The optimized views of all the entitled chromophores are presented in the Fig. 1 and their IUPAC names are recorded in Table S1. The spatial arrangement of atoms in the designed chromophores (**TTBR** and **TTBR-TTB6**) are displayed in Fig. S3, while the Cartesian coordinates are shown in the Tables S2–S8 (Supporting Information).

Various quantum chemical investigations of TTB-based organic chromophores are performed which showed the lower band gaps and significant bathochromic shifts. Moreover, their photovoltaic properties are remarkable and they have sufficient ICT. Thus, the present approach is highly favorable in developing unique organic solar cells (OSCs).

### Electronic analysis

Analysis of FMOs requires how well a molecule promotes charge mobility and electron density distribution^34^. FMOs distribution patterns are helpful in describing the optoelectronic characteristics of the studied compounds (**TTBR** and **TTB1-TTB6**). It also aids in understanding how charge transmission occurs in the OSCs. According to the molecular orbital theory (MOT), the highest occupied molecular orbital (HOMO) is classified as a valence band, whereas the lowest unoccupied molecular orbital (LUMO) is classified as a conduction band. As a result of excitation, electrons move from the valence band (HOMO) to the conduction band (LUMO)^35^. The HOMO/LUMO orbitals are commonly termed as FMOs^36^. The difference between these orbitals is referred to as the band gap (*E*_gap_ = *E*_LUMO_*–E*_HOMO_)^37^ enlisted in the Table S9.

**Fig. 2:**
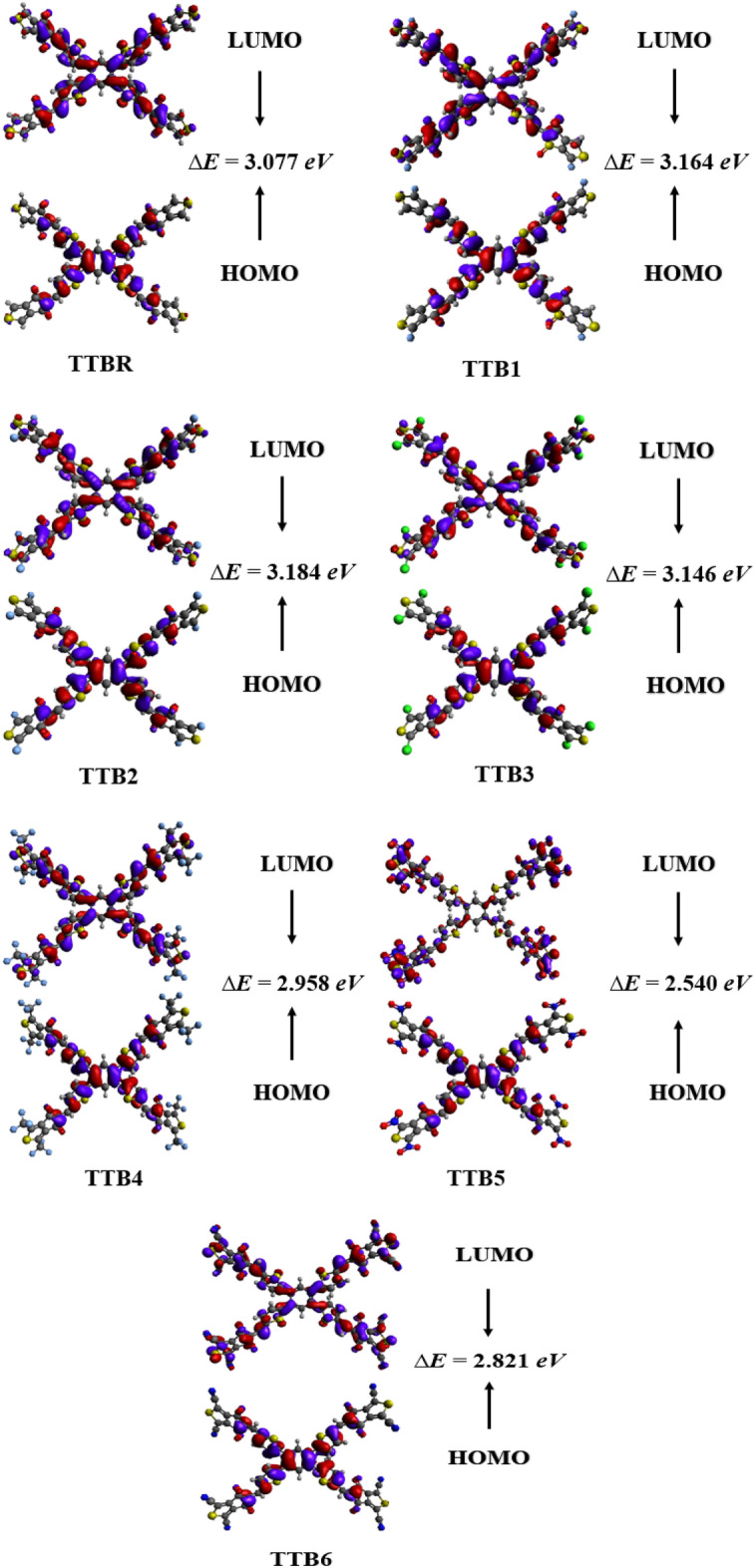
HOMOs and LUMOs of the designed chromophores (**TTBR** and **TTB1-TTB6**).

It is known from the literature that photovoltaic materials with lower bandgaps showed higher power conversion efficiencies (PCEs) and vice versa^38^. The *E*_HOMO_, *E*_LUMO_ and *ΔE* values for **TTBR** reference are noted as − 6.328, − 3.251 and 3.077 eV, respectively. For the designed chromophores **TTB1-TTB6**, the *E*_HOMO_ are − 6.419, − 6.497, − 6.503, − 6.554, − 6.658 and − 6.640 eV, while, the *E*_LUMO_ are − 3.255, − 3.313, − 3.357, − 3.596, − 4.118 and − 3.825 eV, respectively. Similarly, their corresponding Δ*E* are found as 3.164, 3.184, 3.146, 2.958, 2.54 and 2.821 eV, respectively. All the designed chromophores exhibit a decrease in *ΔE* owing to the elevated HOMO and reduced LUMO levels, promoting a significant intramolecular charge transfer (ICT)^39^. Figure 2 depicts a pictorial representation of essential orbitals (HOMO/LUMO). The higher orbitals such as HOMO-1/LUMO + 1 and HOMO-2/LUMO + 2 are also interpreted and their results are recorded in the Table S10, while their orbital surfaces are shown in the Fig. S4.

On comparing the HOMO–LUMO band gaps of the designed derivatives with **TTBR** (3.077 eV), they exhibit lesser energy gaps (2.540–3.184 eV). Among all, the **TTB5** demonstrates the smallest value (2.540 eV). It is a promising candidate in terms of energy band gap owing to the presence of potent electron-withdrawing groups such as nitro (–NO_2_) group on its terminal acceptor moiety (5-methylene-1,3-dinitro-4-*H*-cyclopenta[c]thiophene-4,6(5-*H*)-dione) attached to thiophene rings of the π − spacer. The band gap of **TTB6** (2.821 eV) is lower than that of **TTBR-TTB4** but higher than **TTB5** due to the presence of electron-withdrawing cyano (− CN) groups at terminal sides of 5-methylene-4,6-dioxo-5,6-dihydro-4-*H*-cyclopenta[c]thiophene-1,3-dicarbonitrile acceptor. Further, the **TTB4** (2.958 eV) has a higher band gap than **TTB6** due to the presence of 5-methylene-1,3-bis(trifluoromethyl)-4-*H*-cyclopenta[c]thiophene-4,6(5-*H*)-dione attached to the thiophene rings. Higher band gaps in **TTB1-TTB3** (3.164, 3.184 and 3.146 eV) are due to the presence of less electronegative groups (− F >  − Cl) than (− NO_2_ >  − CN). The largest energy gap observed in **TTB2** might be attributed to the decreased conjugation within the compound. The overall band gaps are found to increase in the following order: **TTB5 < TTB6 < TTB4 < TTBR < TTB3 < TTB1 < TTB2**.

Azeem et al. reported the reference molecule SBDT-BDD has a band gap of 4.49 eV^40^. This band gap is much higher than the current band gap which is in the range of 3.184–2.54 and showed the best photovoltaic properties. The above discussion concluded that **TTB5** exhibits the narrowest Δ*E* among all designed chromophores (**TTBR** and **TTB1-TTB6**) and is the best candidate for photovoltaic OSCs. The majority of the electronic cloud is centered on the π-spacer in HOMO, whereas in the LUMO, it is mostly focused on the over-terminal acceptors and to a lesser extent on the π-spacer. This shows that there is significant facilitation of charge transfer from the π-spacer to the acceptors in all the entitled compounds.

### Global reactivity parameters (GRPs)

The global reactivity descriptors, including ionization potential (*IP*)^41^, electron affinity (*EA*), global hardness (*η*)^42^, chemical potential (*μ*)^43^, electronegativity (*X*)^44^, global softness (*σ*)^45^ and electrophilicity index (*ω*)^46^ are calculated using the Koopman's theorem^47^ listed in Eqs. S1–S5 for **TTBR** and **TTB1-TTB6**. The energy of HOMO determines the *IP*. Similarly, the *EA* reflects the capacity to accept an electron from a donor which is determined by the energy of LUMO. *IP* and *EA* are calculated through Eqs. (1) and (2).

1 $$IP=-{E}_{HOMO},$$

2 $$EA=-{E}_{LUMO},$$

The capability of the compound to absorb additional electrical charge from its surroundings is represented by *ΔN*_max_^48^ and it is computed via Eq. (3).

3 $${\Delta N}_{\text{max}} = -\frac{\mu }{\eta }.$$

A compound with a greater Δ*E* is considered to be hard, exhibiting higher kinetic stability and less reactive. Conversely, a compound with a smaller Δ*E* is found to be softer, highly reactive, and less stable^49^. The ionization energy values of **TTBR** and **TTB1-TTB6** are 6.328, 6.419, 6.497, 6.503, 6.554, 6.658 and 6.646 eV. Similarly, the values for electronegativity and electron affinity are found to be 4.789, 4.837, 4.905, 4.930, 5.075, 5.388, 5.235 eV and 3.251, 3.255, 3.313, 3.357, 3.596, 4.118 and 3.825 *eV,* respectively are listed in the Table 1. The **TTB5** compound exhibits the highest ionization potential (6.658 eV) among all the examined compounds. Moreover, it also showed higher values for *X* = 5.388 and *EA* = 4.118 *eV*. The chemical softness (*σ*) and hardness (*η*) are significant parameters in determining the chemical stability and reactivity. A decreasing trend in *σ* is noticed as follows: **TTB5** (0.393) > **TTB6** (0.354) > **TTB4** (0.338)** > TTBR** (0.324) > **TTB3** (0.317) > **TTB1** (0.316) > **TTB2** (0.314) in *eV*^–1^. Because of the electron-withdrawing characteristics of acceptor moieties, the *σ* values are observed to be smaller than *η* values. The hardness descending trend is as follows: **TTB2** (1.592) > **TTB1** (1.582) > **TTB3** (1.573) > **TTBR** (1.538) > **TTB4** (1.479) > **TTB6** (1.410) > **TTB5** (1.27) in *eV*. Thus, **TTB5** shows the highest *σ* and least *η* values as mentioned in the above trends. Furthermore, **TTB5** exhibits the highest *ΔN*_max_ (4.242 eV). Moreover, the calculated *ΔN*_max_ values decrease in the following sequence in *eV*: **TTB5** (4.242) > **TTB6** (3.712) > **TTB4** (3.431) > **TTB3** (3.134) > **TTBR** (3.113) > **TTB2** (3.081) > **TTB1** (3.057). The unique characteristics observed in **TTB5** due to the presence of –NO_2_ groups on the terminal acceptors, indicate its suitability for the OSCs.

**Table 1:** Ionization potential (*IP*), electron affinity (*EA*), electronegativity (*X*), chemical potential (*μ*), global hardness (*ƞ*), global electrophilicity (ω) and global softness (*σ*) and maximum charge transfer (*ΔN*_*max*_) of the investigated compounds.

Compounds	*IP*	*EA*	*X*	*μ*	*ƞ*	*σ*	*ω*	Δ*N*_max_
TTBR	6.328	3.251	4.789	− 4.789	1.538	0.324	7.455	3.113
TTB1	6.419	3.255	4.837	− 4.837	1.582	0.316	7.394	3.057
TTB2	6.497	3.313	4.905	− 4.905	1.592	0.314	7.556	3.081
TTB3	6.503	3.357	4.930	− 4.930	1.573	0.317	7.725	3.134
TTB4	6.554	3.596	5.075	− 5.075	1.479	0.338	8.707	3.431
TTB5	6.658	4.118	5.388	− 5.388	1.270	0.393	11.42	4.242
TTB6	6.646	3.825	5.235	− 5.235	1.410	0.354	9.716	3.712

Units in *eV* except *σ* (*eV*^−1^).

### Optical analysis

The absorption profile of the organic chromophores employed for organic photovoltaic cells and is an important parameter for determining the efficiency of solar cells^50^. The compounds (**TTBR** and **TTB1-TTB6**) are analyzed by using the TD-DFT computations to investigate their UV–Visible characteristics in dichloromethane solvent. The UV–Visible spectral analysis provides valuable information about electronic transitions in all the investigated molecules and also determines the charge transfer rate^51^. The investigations are conducted to determine the absorption wavelength (*λ*_max_), oscillation strength (*f*_os_), energy of excitation (*E*) and the molecular orbital exhibit a red-shift and lower excitation energies than **TTBR**. Moreover, the literature study shows that molecules with lower transition energy values have higher charge transport ability and may easily undergo excitation between the HOMO and LUMO, resulting in high PCE^32^. A comparative analysis of data in Table S12 indicates that all the compounds exhibit a bathochromic shift in dichloromethane solvent (486.365–605.895 nm) as compared to **TTBR** reference (506.451 nm). The *λ*_max_ values of **TTB1-TTB6** are 490.774, 486.365, 493.509, 526.584, 605.895 and 553.205 nm*,* respectively. The increasing order of *λ*_max_ in the studied chromophores is: **TTB2 < TTB1 < TTB3 < TTBR < TTB4 < TTB6 < TTB5**. This shift towards longer wavelengths is due to solvent effects. The outcomes demonstrate the greater *λ*_max_ values of the designed derivatives are due to strong electron-withdrawing units at their terminal moiety which extended the conjugation. Interestingly, a broader absorption value is obtained for **TTB5** (605.895 nm) relative to other designed chromophores.

The optical properties of compounds are highly affected by internal morphology. Increased crystallinity and optimal molecular arrangement facilitate an increase in conjugation and enhance the efficient overlap of orbitals, resulting in a reduction of the energy difference between the HOMO and LUMO^52^. This promotes effective movement of charges and minimizes losses due to recombination, while internal molecular structures and strong π-π interactions in highly crystalline regions cause a shift towards longer wavelengths (red-shift) in the absorption spectra. This enhances the efficiency of light absorption over a wider range of wavelengths by increasing the electron's mobility and extending conjugation^53^.

**Fig. 3:**
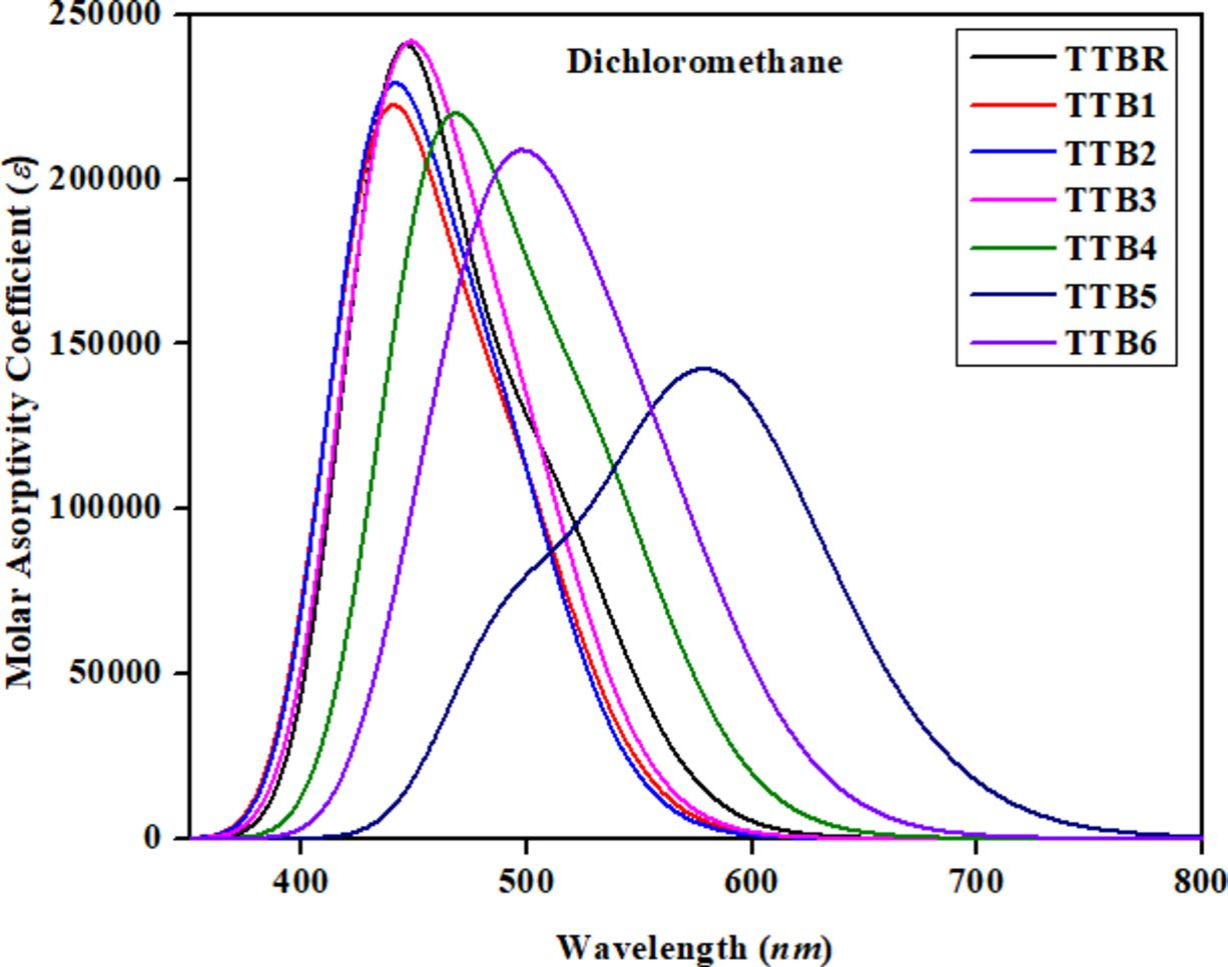
Absorption spectrum of the studied compounds in dichloromethane phases.

The UV–Vis absorption spectra of the studied compounds are depicted in dichloromethane phases shown in the Fig. 3. The higher values of *λ*_max_ (605.895 nm), the lower excitation energy (*E*) 2.046 eV and oscillator strength (*f*_os_) 0.916, with 90% MO contribution from HOMO to LUMO of **TTB5** in dichloromethane is due to the participation of –NO_2_ group at the terminal regions of the compound. The electron-withdrawing group (–NO_2_), effectively pulls the electrons from the π-spacer toward the terminals. As the electron-withdrawing effects of end-capped acceptor groups increase, there is an increase in the *λ*_max_ value, leads to a reduction in HOMO–LUMO band gap. This phenomenon facilitates the charge transfer pathway^54^. From the literature, Azeem et al. reported the optical properties of small molecules with benzodithiophene as a core and the results showed *λ*_max_ 543 nm. While the current study shows *λ*_max_ of 605.895 nm which demonstrated the best optical properties of the designed chromophore.

The transition energy (*E*) demonstrates an inverse correlation with the rate of charge transfer and *λ*_max_^55^. The *E* values of **TTB1-TTB6** are 2.526, 2.549, 2.512, 2.355, 2.046 and 2.241 eV, respectively, whereas the value of **TTBR** is 2.448 eV in the solvent phase listed in the Table S11. The decreasing order of *E* for **TTBR-TTB6** is as follows: **TTB2 > TTB1 > TTB3 > TTBR > TTB4 > TTB6 > TTB5** in *eV*.

Concluding the entire discussion, compounds exhibiting a red shift possess lower energy gaps and increased charge transfer rates, suggesting their excellent photovoltaic response. Thus, they can be utilized as proficient OSCs.

### Density of states (DOS) analysis

**Fig. 4:**
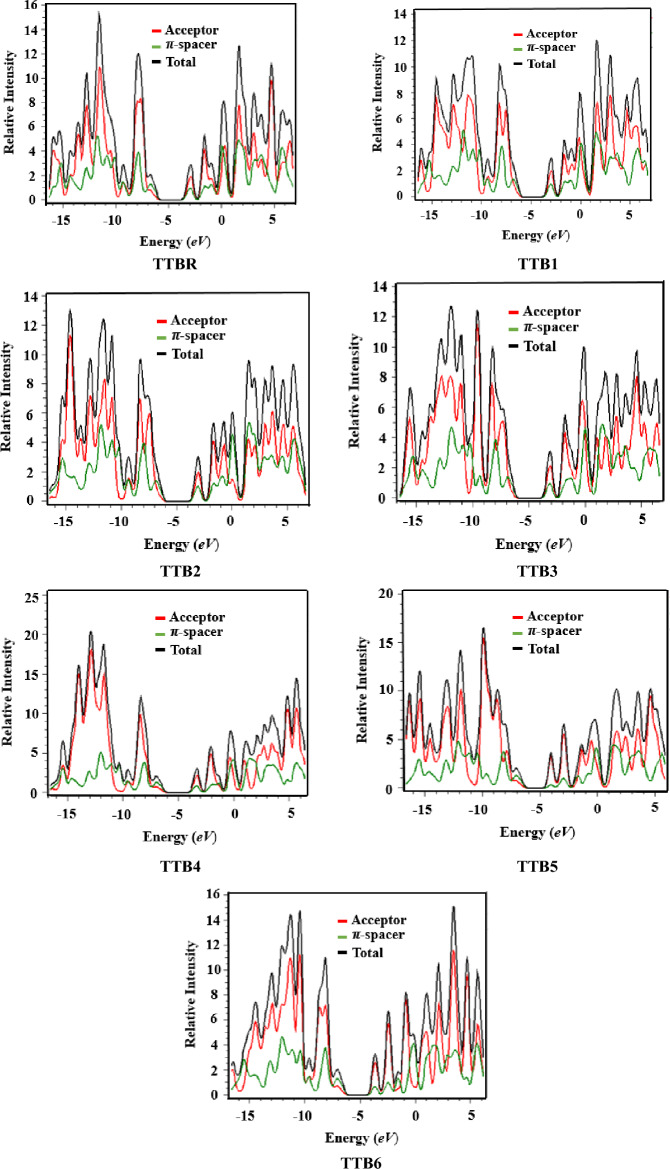
DOS pictographs of the investigated compounds.

Density of states (DOS) is an efficient technique for determining the distribution pattern of electronic density on the FMOs^50^. It is an essential study which aids in investigating the activities of each fragment in the designed (**TTBR** and **TTB1-TTB6**) chromophores i.e. acceptor and π − spacer^54^. It graphically displays the relative intensity on the y-axis and energy over the x-axis. The green colored line shows the π-spacer and the red lines display the acceptor. The x-axis displays the HOMO (valence band) on the right side and the LUMO (conduction band) on the left side in the Fig. 4. The acceptor contributions to LUMO and HOMO in **TTBR** and **TTB1-TTB6** are 56.4, 58.1, 57.6, 60.8, 62.4, 86.0 and 71.0% and 29.4, 29.2, 28.7, 29.4, 29.4, 30.6 and 29.9%, respectively. Similarly, the π-spacer shows 43.6, 41.9, 42.4, 39.2, 37.6, 14.0 and 29.0% for LUMO, while, for HOMO 70.6, 70.8, 71.3, 70.6, 70.6, 69.4 and 70.1% are observed for **TTBR** and **TTB1-TTB6**, respectively as shown in Table S13.

A variety of electron-withdrawing groups are responsible for the different electrical charge distribution patterns in the DOS analysis. The HOMO in these compounds has a reduced electron density on the acceptor and a higher electron density at the π-spacer regions. Contrarily, the LUMO displays a lower electron density on the π-spacer and a larger density on the terminal acceptors. The DOS analysis effectively demonstrates significant charge transfer from the π − bridge towards the peripheral acceptor moieties in all the investigated compounds. Therefore, it is concluded from the DOS plots that end-capped acceptor alteration is a successful method for designing non-fullerene acceptor molecules with excellent optoelectronic and photovoltaic capabilities^1^.

### Transition density matrix (TDM) analysis

**Fig. 5:**
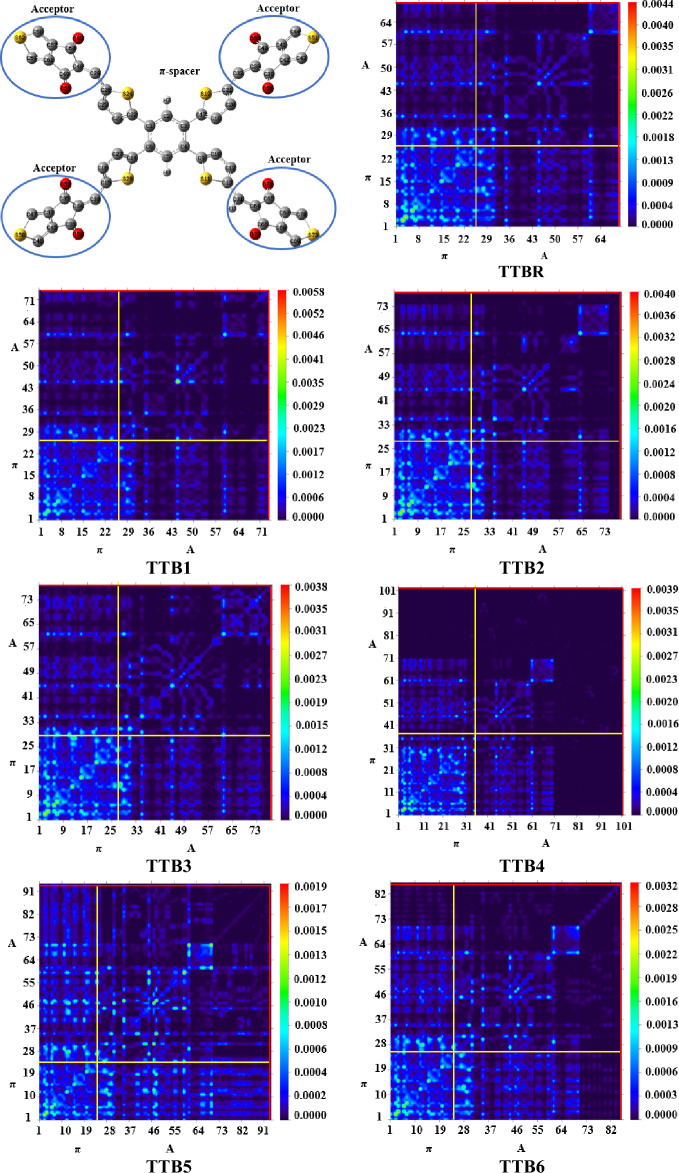
TDM maps of all the investigated chromophores.

Transition density matrix (TDM) analysis plays a key role in the charge transfer and various transitions present within a molecule. It also assists in comprehending some of the characteristics of the neutral state (S_0_) and the excited state (S_1_) transitions. The impact of hydrogen atoms is neglected in all compounds since they have only a little contribution toward the excited state transitions^56^. TDM analysis facilitates the assessment of (i) electronic excitation (ii) localization of the electron holes (iii) interactions between the π-spacer and acceptor moieties in the excited state^57^. For TDM analysis, the designed compounds (**TTBR** and **TTB1-TTB6**) are partitioned into two segments such as the π-spacer (yellow line) and acceptors (red line) as shown in Fig. 5^58^. As per TDM maps, all molecules exhibit coherence in charge distribution. The number of atoms is shown on the x-axis and left y-axis in TDM maps, while, the relative intensity is represented by the right vertical axis. Extended conjugation enhances the charge transfer facilitated via the π-bridge. Subsequently, charge transfer occurs smoothly from one end to another without hindrance. Results show that in all derivatives, the electrical charges are effectively transferred diagonally from the π-spacer to the acceptor components. In compound (**TTB5**), the charge-shift is most noticeable. This phenomenon could arise due to the potent electron-withdrawing nitro (–NO_2_) groups at the terminal side acceptors. Thus, incorporating end-capped electron-withdrawing acceptors in the newly designed organic chromophores improves the electron transport from the π-spacer to the acceptor regions, making them efficient for OSC applications.

### Exciton binding energy (E_b_)

Binding energy (*E*_b_) is another factor for studying the optoelectronic properties and excitation dissociation potential in the OSCs. It is known as the Columbic interactions between negative and positive charges^56^. The binding energy can be determined by subtracting the minimum energy required for the first excitation (*E*_opt_) from the energy gap between the HOMO–LUMO^59^. Therefore, a lower value of exciton binding energy is correlated with a weaker electron–hole interaction and an increase in charge transfer^57^. Lower values of *E*_b_ provide high current charge density (*J*_*sc*_), high charge dissociation and high power conversion efficiency^60^. The Eq. (4) is used to calculate the binding energy^61^.

4 $$E_{{\text{b}}} = \, E_{{{\text{gap}}}} - E_{{{\text{opt}}}} ,$$

Here, the *E*_b_ represents binding energy, *E*_gap_ is the energy gap and *E*_opt_ corresponds to the single-point energy.

The results obtained are listed in Table 2. The highest value of *E*_b_ is 0.638 eV in the case of **TTB1**. However, the lowest value is shown by **TTB5** (0.494 eV), indicating the highest levels of charge dissociation and rate of charge transfer**.** The decreasing trend of exciton binding energy is **TTB1 > TTB2 > TTB3 > TTBR > TTB4 > TTB6 > TTB5**. *E*_b_ is essential for determining both the efficiency and functionality of photovoltaic cells. Decreased *E*_b_ enhanced photovoltaic characteristics, such as increased charge separation efficiency, greater *V*_OC_ and improved overall energy conversion efficiency.

**Table 2:** Computed binding energies of **TTBR** and **TTB1-TTB6**.

Compounds	*E*_H-L_	*E*_opt_	*E*_*b*_
TTBR	3.077	2.448	0.629
TTB1	3.164	2.526	0.638
TTB2	3.184	2.549	0.635
TTB3	3.146	2.512	0.634
TTB4	2.958	2.355	0.603
TTB5	2.54	2.046	0.494
TTB6	2.821	2.241	0.580

Units in *eV*.

### Hole-electron analysis

**Fig. 6:**
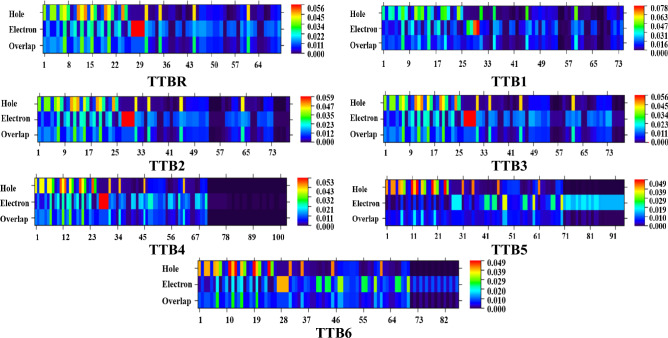
Pictorial illustration of the hole-electron analysis for the titled chromophores.

When an excited electron in the hole region migrates to the electron region, this phenomenon is known as hole-electron analysis. In OSCs, the charge transfer (CT) state occurs at the interface between π-spacer and electron acceptor. This CT state is essential for the separation of charges^62^. This analysis is highly effective and widely utilized for identifying the localization of electron density within a compound^63^. It is a feasible approach for revealing the nature of electron excitations and charge transfer^64^. The designed chromophores possess tetra thienyl benzene as the π-spacer and electron-withdrawing acceptors at terminals. Moreover, it is observed that in the reference (**TTBR**) and designed compounds (**TTB2-TTB4)**, the hole arises from a carbon atom within the thiophene ring (π-spacer) and the electron density is maximum in the electron band at the carbon atom in between the terminal acceptor groups and π-spacer. But in **TTB1**, the hole band has medium density and in the electron band, maximum intensity is same as mentioned for the above designed chromophores. Notably, all pictographs demonstrate that the hole emerges within the π-linker segment at various atoms, while the electron intensity reaches its peak at the carbon atom of the π-linker and acceptor as demonstrated in the Fig. 6. Moreover, in **TTB5** and **TTB6**, the electron intensity is high in the hole band at C-24. The results indicate that **TTBR-TTB4** are observed as electron-type material, whereas **TTB5-TTB6** compounds demonstrate themselves as hole-type materials.

## Conclusion

In summary, a series of A-π-A non-fullerene acceptor molecules (**TTBR** and **TTB1-TTB7**) are designed to explore their photovoltaic properties for OSC applications. Quantum chemical analysis is performed to analyze their photovoltaic, photophysical and electronic characteristics. All designed chromophores have demonstrated favorable outcomes for various computational analyses. Among all the designed compounds, **TTB5** displayed the least band gap value of 2.540 eV and the bathochromic shift (*λ*_max_ = 605.895 nm), highest softness (0.393 eV^*−1*^) and lowest hardness (1.27 eV). The decreasing trend of *X* is **TTB5 > TTB6 > TTB4 > TTB3 > TTB2 > TTB1 > TTBR**. The *E*_b_ of **TTBR-TTB6** is comparable among them, leading to higher exciton dissociation and lower binding energy (*E*_b_ = 0.494–0.638 eV). The results from the analysis of all geometrical parameters demonstrate that modifications of end-capped acceptors represent highly efficient photovoltaic materials with superior optoelectronic properties. In short, it is clear that all afore-mentioned compounds are obtained by using structural modifications and they show potential for OSCs.

## Supplementary Information

Supplementary Information.

## Data Availability

All data generated or analyzed during this study are included in this published article and its supplementary information files.
