# Supplementary material for: Unveiling peripheral symmetric acceptors coupling with tetrathienylbenzene core to promote electron transfer dynamics in organic photovoltaics
Source: Sci Rep. 2024 Sep 11;14:21176. doi: 10.1038/s41598-024-71777-6 (PMC11387658; doi:10.1038/s41598-024-71777-6)
Supplement: Supplementary file 1 — Supplementary Information. [file 41598_2024_71777_MOESM1_ESM.docx]

**Supplementary data**

**Unveiling Peripheral Symmetric Acceptors Coupling with Tetrathienylbenzene Core to Promote Electron Transfer Dynamics in Organic Photovoltaics**

Muhammad Khalid,^a,b^* Aiman Jabbar,^a,b^ Shahzad Murtaza,^a,b^ Muhammad Arshad,^c^ Ataualpa A. C. Braga,^d^ Tansir Ahamad,^e^

^a^Institute of Chemistry, Khwaja Fareed University of Engineering & Information Technology, Rahim Yar Khan, 64200, Pakistan

^b^Centre for Theoretical and Computational Research, Khwaja Fareed University of Engineering & Information Technology, Rahim Yar Khan, 64200, Pakistan

^c^Industry Solutions, Northern Alberta Institute of Technology, Edmonton, Alberta, Canada

^d^Departamento de Química Fundamental, Instituto de Química, Universidade de São Paulo, Av. Prof. Lineu Prestes, 748, São Paulo, 05508-000, Brazil

^e^Department of Chemistry, College of Science, King Saud University, Saudi Arabia

*Corresponding author E-mail addresses:

Dr. Muhammad Khalid: ([khalid@iq.usp.br](mailto:khalid@iq.usp.br) ; [muhammad.khalid@kfueit.edu.pk](mailto:muhammad.khalid@kfueit.edu.pk))

**Table S1**: The IUPAC names of **TTBR** and its derivatives (**TTB1-TTB6**).

| **TTBR** | 5,5',5'',5'''-((5,5',5'',5'''-(benzene-1,2,4,5-tetrayl)tetrakis(thiophene-5,2-diyl))tetrakis(methanylylidene))tetrakis(4-*H*-cyclopenta[c]thiophene-  4,6(5-*H*)-dione) compound with methane (1:2) |
| --- | --- |
| **TTB1** | 5,5',5'',5'''-((5,5',5'',5'''-(benzene-1,2,4,5-tetrayl)tetrakis(thiophene-5,2-diyl))tetrakis(methanylylidene))tetrakis(1-fluoro-4-*H*-cyclopenta[c]  thiophene-4,6(5-*H*)-dione) compound with methane (1:2) |
| **TTB2** | 5,5',5'',5'''-((5,5',5'',5'''-(benzene-1,2,4,5-tetrayl)tetrakis(thiophene-5,2-diyl))tetrakis(methanylylidene))tetrakis(1,3-difluoro-4-*H*-cyclopenta[c]  thiophene-4,6(5-*H*)-dione) compound with methane (1:2) |
| **TTB3** | 5,5',5'',5'''-((5,5',5'',5'''-(benzene-1,2,4,5-tetrayl)tetrakis(thiophene-5,2-diyl))tetrakis(methanylylidene))tetrakis(1,3-dichloro-4-*H*-cyclopenta[c]  thiophene-4,6(5-*H*)-dione) compound with methane (1:2) |
| **TTB4** | 5,5',5'',5'''-((5,5',5'',5'''-(benzene-1,2,4,5-tetrayl)tetrakis(thiophene-5,2 diyl))tetrakis(methanylylidene))tetrakis(1,3-bis(trifluoromethyl)-4*H*-cyclopenta[c]thiophene-4,6(5-*H*)-dione) compound with methane (1:2) |
| **TTB5** | 5,5',5'',5'''-((5,5',5'',5'''-(benzene-1,2,4,5-tetrayl)tetrakis(thiophene-5,2-diyl))tetrakis(methanylylidene))tetrakis(1,3-dinitro-4-*H*-cyclopenta[c]  thiophene-4,6(5-*H*)-dione) compound with methane (1:2) |
| **TTB6** | 5,5',5'',5'''-((5,5',5'',5'''-(benzene-1,2,4,5-tetrayl)tetrakis(thiophene-5,2-diyl))tetrakis(methanylylidene))tetrakis(4,6-dioxo-5,6-dihydro-4-*H*-  cyclopenta[c]thiophene-1,3-dicarbonitrile) compound with methane (1:2) |

**Table S2**: Cartesian Coordinates of **TTBR** compound.

| **Atom** | **X-axis** | **Y-axis** | **Z-axis** |
| --- | --- | --- | --- |
| C | -1.22053 | -0.704214 | 0.036083 |
| C | 0.000148 | -1.372412 | 0.000018 |
| C | 1.220798 | -0.704183 | -0.036023 |
| C | 1.220761 | 0.704402 | 0.036045 |
| C | 0.000089 | 1.372587 | 0.000002 |
| C | -1.220567 | 0.704341 | -0.036017 |
| H | 0.000133 | -2.459243 | -0.000004 |
| H | 0.000058 | 2.459418 | -0.000023 |
| C | 2.456255 | 1.471408 | 0.2017 |
| C | 3.484821 | 1.21202 | 1.081982 |
| S | 2.761139 | 2.903204 | -0.70801 |
| C | 4.512111 | 2.156703 | 1.034976 |
| H | 3.475292 | 0.359572 | 1.75203 |
| C | 4.289635 | 3.160555 | 0.109052 |
| H | 5.403397 | 2.143986 | 1.648189 |
| C | -2.456094 | 1.471281 | -0.201729 |
| C | -4.289495 | 3.160427 | -0.109238 |
| C | -4.511825 | 2.15664 | -1.035287 |
| C | 2.45631 | -1.471145 | -0.201636 |
| C | 3.485092 | -1.211542 | -1.081618 |
| S | 2.760938 | -2.903231 | 0.70769 |
| C | 4.512334 | -2.156268 | -1.034668 |
| H | 3.475735 | -0.358884 | -1.751397 |
| C | 4.289609 | -3.160407 | -0.109089 |
| H | 5.403831 | -2.143427 | -1.647583 |
| C | -2.456016 | -1.471214 | 0.201842 |
| C | -3.484311 | -1.212086 | 1.082506 |
| S | -2.76117 | -2.902747 | -0.7082 |
| C | -4.51166 | -2.156714 | 1.03548 |
| H | -3.474592 | -0.359772 | 1.752726 |
| C | -4.289446 | -3.16032 | 0.109217 |
| C | -3.484507 | 1.211987 | -1.082232 |
| S | -2.761132 | 2.902995 | 0.708045 |
| C | -5.057256 | -4.296487 | -0.265203 |
| C | -5.057277 | 4.296628 | 0.265132 |
| C | 5.057334 | 4.296841 | -0.265243 |
| C | 5.057102 | -4.296876 | 0.265049 |
| H | -4.608216 | 4.930701 | 1.034983 |
| H | 4.608228 | 4.930937 | -1.035053 |
| H | 4.607747 | -4.931136 | 1.034573 |
| H | -4.608307 | -4.930419 | -1.035237 |
| C | -6.264136 | 4.760987 | -0.149459 |
| C | -7.231996 | 4.251043 | -1.148867 |
| C | -6.800405 | 6.009376 | 0.46522 |
| C | -8.358692 | 5.207195 | -1.118633 |
| C | 6.263952 | -4.761351 | -0.149456 |
| C | 7.232108 | -4.2513 | -1.148519 |
| C | 6.799785 | -6.010107 | 0.46485 |
| C | 8.108883 | -6.238187 | -0.175675 |
| C | 8.358591 | -5.207703 | -1.118384 |
| C | 9.549053 | -5.337165 | -1.760628 |
| H | 9.996234 | -4.709135 | -2.518754 |
| C | 9.108343 | -7.155573 | -0.09728 |
| H | 9.189776 | -8.035872 | 0.524986 |
| C | -8.109414 | 6.237397 | -0.175507 |
| C | -9.109078 | 7.154556 | -0.097023 |
| H | -9.190822 | 8.034622 | 0.525531 |
| C | -9.549026 | 5.336647 | -1.761119 |
| H | -9.995902 | 4.708798 | -2.519575 |
| C | -6.264058 | -4.760933 | 0.149458 |
| C | -7.231757 | -4.251239 | 1.149149 |
| S | -10.374669 | 6.745474 | -1.198254 |
| S | 10.374266 | -6.746373 | -1.198089 |
| O | -7.15311 | -3.268266 | 1.859122 |
| O | -7.153461 | 3.267892 | -1.858606 |
| O | -6.261611 | 6.685686 | 1.314524 |
| O | 6.260634 | -6.686633 | 1.313756 |
| O | 7.153949 | -3.267867 | -1.857911 |
| C | -8.109348 | -6.237324 | 0.175397 |
| C | -9.10908 | -7.154394 | 0.096751 |
| H | -9.190906 | -8.034318 | -0.525993 |
| C | -9.548824 | -5.336826 | 1.761278 |
| H | -9.995625 | -4.709121 | 2.519895 |
| C | -6.800374 | -6.00923 | -0.465367 |
| C | -8.358509 | -5.207319 | 1.118774 |
| S | -10.374595 | -6.745467 | 1.198129 |
| O | -6.261602 | -6.685472 | -1.314739 |
| C | 6.264148 | 4.761278 | 0.149364 |
| C | 7.232029 | 4.251419 | 1.148811 |
| C | 6.800291 | 6.009769 | -0.465239 |
| C | 8.358649 | 5.207655 | 1.118585 |
| C | 8.109278 | 6.237867 | 0.175489 |
| C | 9.108894 | 7.155077 | 0.09698 |
| H | 9.190577 | 8.03516 | -0.525558 |
| C | 9.549 | 5.337152 | 1.761024 |
| H | 9.995941 | 4.709316 | 2.519451 |
| O | 7.153609 | 3.268187 | 1.858441 |
| O | 6.261448 | 6.686058 | -1.314528 |
| S | 10.374533 | 6.74605 | 1.198174 |
| H | -3.474857 | 0.359546 | -1.752293 |
| H | -5.402785 | -2.144209 | 1.648934 |
| H | -5.403087 | 2.143974 | -1.648545 |

**Table S3**: Cartesian Coordinates of **TTB1** compound.

| **Atom** | **X-axis** | **Y-axis** | **Z-axis** |
| --- | --- | --- | --- |
| C | -1.17354 | -0.47817 | -0.32564 |
| C | 0.012135 | -1.20773 | -0.34438 |
| C | 1.267039 | -0.60536 | -0.33589 |
| C | 1.343118 | 0.800199 | -0.28322 |
| C | 0.157701 | 1.530328 | -0.30825 |
| C | -1.0969 | 0.928239 | -0.34122 |
| H | -0.04251 | -2.29298 | -0.35141 |
| H | 0.212057 | 2.615379 | -0.28658 |
| C | 2.616909 | 1.508304 | -0.13967 |
| C | 3.641066 | 1.229514 | 0.740576 |
| S | 2.97139 | 2.903381 | -1.08635 |
| C | 4.702397 | 2.133771 | 0.666353 |
| C | 4.512276 | 3.123294 | -0.28276 |
| H | 5.595003 | 2.102782 | 1.276908 |
| C | -2.29062 | 1.777008 | -0.39071 |
| C | -4.01958 | 3.522134 | 0.026201 |
| C | -4.33063 | 2.682335 | -1.02906 |
| C | 2.461467 | -1.45335 | -0.38215 |
| C | 3.545171 | -1.36076 | -1.22763 |
| S | 2.633532 | -2.7682 | 0.71596 |
| C | 4.51199 | -2.34612 | -1.00394 |
| H | 3.621031 | -0.59724 | -1.99391 |
| C | 4.183198 | -3.20687 | 0.028783 |
| H | 5.43284 | -2.46219 | -1.56196 |
| C | -2.45051 | -1.18853 | -0.22703 |
| C | -3.49135 | -0.93594 | 0.638505 |
| S | -2.7911 | -2.55012 | -1.22752 |
| C | -4.55496 | -1.8344 | 0.512604 |
| H | -3.46992 | -0.12133 | 1.354003 |
| C | -4.34722 | -2.79349 | -0.46122 |
| H | -5.46088 | -1.81266 | 1.10218 |
| C | -3.35984 | 1.701817 | -1.25631 |
| S | -2.48185 | 3.069434 | 0.730675 |
| C | -5.14278 | -3.8779 | -0.94041 |
| C | -4.70096 | 4.622255 | 0.611505 |
| C | 5.317248 | 4.220656 | -0.68975 |
| C | 4.85571 | -4.31746 | 0.604908 |
| H | -4.18641 | 5.090209 | 1.455627 |
| H | 4.328715 | -4.79996 | 1.432997 |
| H | -4.68823 | -4.41013 | -1.78057 |
| C | -5.88676 | 5.222222 | 0.334895 |
| C | -6.91261 | 4.956107 | -0.6975 |
| C | -6.30982 | 6.377288 | 1.176315 |
| C | -7.9672 | 5.970127 | -0.46803 |
| C | 6.045695 | -4.91231 | 0.335695 |
| C | 7.085362 | -4.62957 | -0.67825 |
| C | 6.457486 | -6.08101 | 1.163993 |
| C | 7.764375 | -6.49386 | 0.628929 |
| C | 8.136984 | -5.64701 | -0.45077 |
| C | 9.331945 | -5.94767 | -1.01483 |
| H | 9.84632 | -5.47164 | -1.83789 |
| C | 8.703796 | -7.4383 | 0.865886 |
| C | -7.60914 | 6.799221 | 0.630121 |
| C | -8.55173 | 7.739663 | 0.870169 |
| C | -9.15458 | 6.280172 | -1.04292 |
| H | -9.65817 | 5.817741 | -1.88032 |
| C | -6.33109 | -4.38578 | -0.5377 |
| C | -7.20013 | -4.04265 | 0.603587 |
| S | -9.88833 | 7.630266 | -0.23886 |
| S | 10.05518 | -7.31069 | -0.22318 |
| S | -6.89456 | -5.85558 | -1.49581 |
| O | -7.10811 | -3.06624 | 1.322602 |
| O | -6.92303 | 4.103585 | -1.56271 |
| O | -5.7036 | 6.856725 | 2.108948 |
| O | 5.838466 | -6.57553 | 2.080123 |
| O | 7.106923 | -3.76381 | -1.52996 |
| F | -8.55775 | 8.667363 | 1.797956 |
| F | 8.69732 | -8.38181 | 1.777587 |
| C | -8.13684 | -6.1214 | -0.22198 |
| C | -9.08258 | -7.06649 | -0.03132 |
| C | -9.2022 | -5.27652 | 1.668091 |
| H | -9.47048 | -4.65035 | 2.507652 |
| C | 6.535476 | 4.662453 | -0.2816 |
| C | 7.109854 | 5.873152 | -0.93481 |
| C | 7.474573 | 4.160016 | 0.745968 |
| C | 8.629069 | 5.084424 | 0.703245 |
| C | 8.413393 | 6.085143 | -0.2832 |
| C | 9.454829 | 6.946779 | -0.34903 |
| C | 9.806202 | 5.189499 | 1.366181 |
| H | 10.21409 | 4.569502 | 2.15227 |
| H | 4.892501 | 4.842256 | -1.48315 |
| F | 9.605629 | 7.985936 | -1.13615 |
| F | -9.30809 | -8.15016 | -0.73928 |
| C | -8.21033 | -5.09717 | 0.754344 |
| O | -5.83258 | -6.91192 | -1.42169 |
| O | 6.600205 | 6.532422 | -1.81392 |
| O | 7.363553 | 3.203108 | 1.486134 |
| S | -10.0749 | -6.73085 | 1.358733 |
| S | 10.71135 | 6.551257 | 0.788114 |
| H | -3.42283 | 0.953114 | -2.03825 |
| H | -5.24225 | 2.809566 | -1.5996 |
| H | 3.604319 | 0.395298 | 1.432227 |

**Table S4:** Cartesian Coordinates of **TTB2** compound.

| **Atom** | **X-axis** | **Y-axis** | **Z-axis** |
| --- | --- | --- | --- |
| C | -1.22286 | 0.703499 | 0.002396 |
| C | -2E-05 | 1.369537 | -0.00023 |
| C | 1.222852 | 0.70358 | -0.003 |
| C | 1.222923 | -0.7036 | 0.002256 |
| C | 8.5E-05 | -1.36963 | -0.0005 |
| C | -1.22279 | -0.70367 | -0.00313 |
| H | -4.1E-05 | 2.455956 | -8.7E-05 |
| H | 0.000104 | -2.45605 | -0.00055 |
| C | 2.457033 | -1.49375 | 0.026724 |
| C | 3.539422 | -1.38154 | 0.872815 |
| S | 2.669577 | -2.78182 | -1.0953 |
| C | 4.533128 | -2.33622 | 0.634657 |
| C | 4.228269 | -3.19018 | -0.41162 |
| C | -2.45686 | -1.4939 | -0.02753 |
| C | -4.22832 | -3.18993 | 0.411339 |
| C | -4.53318 | -2.33609 | -0.63504 |
| C | 2.456916 | 1.493813 | -0.02737 |
| C | 3.539232 | 1.38177 | -0.87357 |
| S | 2.669571 | 2.781551 | 1.094981 |
| C | 4.533025 | 2.336306 | -0.63517 |
| C | 4.228274 | 3.190004 | 0.411352 |
| C | -2.45698 | 1.493657 | 0.02692 |
| C | -3.53933 | 1.381488 | 0.873067 |
| S | -2.66963 | 2.781565 | -1.09526 |
| C | -4.53312 | 2.336053 | 0.634784 |
| C | -4.22831 | 3.189945 | -0.41157 |
| C | -3.5393 | -1.38167 | -0.87355 |
| S | -2.66949 | -2.78169 | 1.094774 |
| C | -4.92347 | 4.278507 | -0.99982 |
| C | -4.92343 | -4.27847 | 0.999689 |
| C | 4.923328 | -4.27891 | -0.9997 |
| C | 4.923382 | 4.278535 | 0.999713 |
| H | -4.4127 | -4.75168 | 1.843252 |
| H | 4.41266 | 4.751723 | 1.843298 |
| H | -4.41283 | 4.751728 | -1.84344 |
| C | -6.11415 | -4.87047 | 0.724325 |
| C | -7.13073 | -4.59884 | -0.31461 |
| C | -6.5393 | -6.02522 | 1.562037 |
| C | -8.1802 | -5.61733 | -0.09677 |
| C | 6.114058 | 4.870594 | 0.724304 |
| C | 7.130714 | 4.598847 | -0.31452 |
| C | 6.539173 | 6.02541 | 1.56194 |
| C | 7.833275 | 6.455246 | 1.006405 |
| C | 8.180187 | 5.617347 | -0.09671 |
| C | 9.357992 | 5.965166 | -0.65341 |
| C | 8.757757 | 7.40632 | 1.24701 |
| C | -7.83338 | -6.4551 | 1.006473 |
| C | -8.75792 | -7.4061 | 1.247156 |
| C | -9.35801 | -5.96515 | -0.65347 |
| C | -6.11413 | 4.870541 | -0.72428 |
| C | -7.1306 | 4.598884 | 0.314758 |
| S | -10.1068 | -7.33408 | 0.137169 |
| S | 10.10665 | 7.334276 | 0.137038 |
| O | -7.13468 | 3.741043 | 1.17352 |
| O | -7.13488 | -3.74105 | -1.17342 |
| O | -5.94073 | -6.50548 | 2.49887 |
| O | 5.9406 | 6.50569 | 2.498768 |
| O | 7.134974 | 3.7409 | -1.17319 |
| H | -5.45536 | -2.43666 | -1.19401 |
| F | -8.7546 | -8.33157 | 2.175508 |
| F | 8.754334 | 8.331908 | 2.175239 |
| F | -9.97518 | -5.41907 | -1.67345 |
| F | 9.975249 | 5.418989 | -1.6733 |
| C | -7.83341 | 6.455169 | -1.00621 |
| C | -8.75802 | 7.406118 | -1.24682 |
| C | -9.35793 | 5.965091 | 0.653801 |
| S | -10.1068 | 7.334006 | -0.13675 |
| O | -5.94087 | 6.50566 | -2.49875 |
| C | -6.53935 | 6.025355 | -1.56187 |
| C | 6.113957 | -4.87093 | -0.72407 |
| C | 7.130531 | -4.599 | 0.314805 |
| C | 6.539155 | -6.02594 | -1.56143 |
| C | 7.833376 | -6.45539 | -1.00595 |
| C | 8.1802 | -5.61725 | 0.09702 |
| C | 9.35825 | -5.96458 | 0.653512 |
| C | 8.758105 | -7.40623 | -1.24656 |
| H | 4.412628 | -4.75227 | -1.8432 |
| O | 5.940521 | -6.50658 | -2.49803 |
| O | 7.134679 | -3.7409 | 1.173319 |
| S | 10.1072 | -7.33355 | -0.13689 |
| H | 3.592718 | 0.632635 | -1.65575 |
| H | 3.593025 | -0.63217 | 1.654767 |
| H | -3.59286 | 0.632147 | 1.655043 |
| H | -3.59286 | -0.63236 | -1.65556 |
| H | -5.45517 | 2.436718 | 1.193934 |
| H | 5.455251 | -2.4368 | 1.19371 |
| H | 5.455122 | 2.436982 | -1.19425 |
| F | -9.97501 | 5.41896 | 1.673815 |
| F | -8.75482 | 8.331601 | -2.17515 |
| F | 9.975511 | -5.41809 | 1.673224 |
| F | 8.754817 | -8.33193 | -2.17468 |
| C | -8.18012 | 5.617364 | 0.097033 |

**Table S5:** Cartesian Coordinates of **TTB3** compound.

| **Atom** | **X-axis** | **Y-axis** | **Z-axis** |
| --- | --- | --- | --- |
| C | -1.22293 | -0.70391 | -0.00445 |
| C | -0.00035 | -1.37027 | -0.00011 |
| C | 1.222155 | -0.70381 | 0.004249 |
| C | 1.222088 | 0.703528 | -0.00449 |
| C | -0.00046 | 1.36991 | -2.6E-05 |
| C | -1.223 | 0.703466 | 0.004296 |
| H | -0.00028 | -2.45671 | -0.00011 |
| H | -0.00044 | 2.456355 | 0.000105 |
| C | 2.457117 | 1.491896 | -0.03568 |
| C | 3.535272 | 1.373029 | -0.88627 |
| S | 2.680032 | 2.78159 | 1.082585 |
| C | 4.535223 | 2.32193 | -0.65306 |
| C | 4.239308 | 3.179134 | 0.393321 |
| C | -2.45803 | 1.491836 | 0.035296 |
| C | -4.23969 | 3.179693 | -0.39358 |
| C | -4.53659 | 2.3215 | 0.651705 |
| C | 2.457253 | -1.49207 | 0.035375 |
| C | 3.535578 | -1.37295 | 0.885733 |
| S | 2.680084 | -2.78192 | -1.08272 |
| C | 4.535528 | -2.32185 | 0.652552 |
| C | 4.239419 | -3.17931 | -0.39358 |
| C | -2.45791 | -1.49234 | -0.0354 |
| C | -3.53698 | -1.37242 | -0.88468 |
| S | -2.67987 | -2.78317 | 1.08178 |
| C | -4.53672 | -2.32157 | -0.65157 |
| C | -4.23979 | -3.17996 | 0.393542 |
| C | -3.537 | 1.372212 | 0.884764 |
| S | -2.67995 | 2.782597 | -1.08199 |
| C | -4.94662 | -4.26275 | 0.978195 |
| C | -4.94658 | 4.262373 | -0.97838 |
| C | 4.946566 | 4.261398 | 0.978435 |
| C | 4.94673 | -4.26156 | -0.97864 |
| H | -4.43946 | 4.74752 | -1.81726 |
| H | 4.44007 | -4.74633 | -1.81801 |
| H | -4.43937 | -4.74813 | 1.816855 |
| C | -6.14903 | 4.831701 | -0.70496 |
| C | -7.16799 | 4.534045 | 0.322523 |
| C | -6.5972 | 5.978794 | -1.53954 |
| C | -8.24509 | 5.524137 | 0.094059 |
| C | 6.149176 | -4.83078 | -0.70501 |
| C | 7.167607 | -4.53342 | 0.323061 |
| C | 6.597973 | -5.97733 | -1.54001 |
| C | 7.913645 | -6.36826 | -0.99819 |
| C | 8.245218 | -5.52291 | 0.094297 |
| C | 9.445855 | -5.81973 | 0.650237 |
| C | 8.864033 | -7.29827 | -1.26346 |
| C | -7.91287 | 6.369952 | -0.99787 |
| C | -8.86269 | 7.300622 | -1.2628 |
| C | -9.4457 | 5.821258 | 0.649898 |
| C | -6.14917 | -4.8319 | 0.704878 |
| C | -7.16825 | -4.5339 | -0.32239 |
| C | -7.91313 | -6.37002 | 0.997716 |
| S | -10.2078 | 7.162735 | -0.16172 |
| S | 10.20883 | -7.16034 | -0.16198 |
| O | -7.16216 | -3.67754 | -1.18254 |
| O | -7.1619 | 3.677813 | 1.182805 |
| O | -6.00379 | 6.482686 | -2.46689 |
| O | 6.005025 | -6.48092 | -2.46782 |
| O | 7.160896 | -3.6777 | 1.183846 |
| H | -5.45793 | 2.414504 | 1.213423 |
| Cl | -10.22 | 5.062426 | 1.970863 |
| Cl | -8.86078 | 8.496995 | -2.4811 |
| Cl | 8.863124 | -8.49402 | -2.48237 |
| Cl | 10.21955 | -5.06129 | 1.9718 |
| C | -8.24541 | -5.52396 | -0.09402 |
| C | -9.44617 | -5.82078 | -0.64967 |
| C | -8.86312 | -7.30051 | 1.262723 |
| C | 6.149033 | 4.830681 | 0.704972 |
| C | 6.597781 | 5.977114 | 1.540126 |
| C | 7.167578 | 4.533394 | -0.32304 |
| C | 8.245071 | 5.523005 | -0.09423 |
| C | 7.913445 | 6.368171 | 0.998395 |
| C | 8.863693 | 7.298301 | 1.263746 |
| C | 9.44565 | 5.820094 | -0.65015 |
| H | 4.439807 | 4.746095 | 1.81779 |
| C | -6.59736 | -5.97911 | 1.539294 |
| O | -6.00393 | -6.48318 | 2.466539 |
| O | 7.160941 | 3.677809 | -1.18395 |
| O | 6.004766 | 6.48061 | 2.467956 |
| S | 10.20842 | 7.160781 | 0.162114 |
| S | -10.2084 | -7.16225 | 0.161859 |
| Cl | 10.21943 | 5.061883 | -1.9718 |
| Cl | 8.862621 | 8.49374 | 2.482961 |
| Cl | -10.2206 | -5.0616 | -1.97035 |
| Cl | -8.86127 | -8.49705 | 2.480862 |
| H | 3.58178 | 0.622357 | -1.6674 |
| H | 3.582207 | -0.62201 | 1.666589 |
| H | 5.456557 | -2.41515 | 1.214755 |
| H | 5.456125 | 2.415316 | -1.21544 |
| H | -3.58431 | 0.620804 | 1.665122 |
| H | -3.58433 | -0.62098 | -1.66499 |
| H | -5.45819 | -2.41429 | -1.21312 |

**Table S6:** Cartesian Coordinates of **TTB4** compound.

| **Atom** | **X-axis** | **Y-axis** | **Z-axis** |
| --- | --- | --- | --- |
| C | 1.22072 | 0.704169 | 0.033962 |
| C | 4.3E-05 | 1.372364 | -6E-05 |
| C | -1.22063 | 0.704167 | -0.03404 |
| C | -1.22063 | -0.70415 | 0.033994 |
| C | 4.2E-05 | -1.37235 | 8.99E-06 |
| C | 1.22072 | -0.70415 | -0.03401 |
| H | 4.11E-05 | 2.458932 | -7.9E-05 |
| H | 4.1E-05 | -2.45891 | 2.8E-05 |
| C | -2.45396 | -1.4746 | 0.197304 |
| C | -3.48395 | -1.21962 | 1.080635 |
| S | -2.74222 | -2.91265 | -0.7031 |
| C | -4.49484 | -2.17923 | 1.04771 |
| H | -3.4815 | -0.36379 | 1.746072 |
| C | -4.25982 | -3.18934 | 0.12812 |
| H | -5.38027 | -2.17465 | 1.668907 |
| C | 2.45404 | -1.47462 | -0.19731 |
| C | 4.259882 | -3.18937 | -0.12808 |
| C | 4.494881 | -2.17932 | -1.04774 |
| C | -2.45396 | 1.474618 | -0.19735 |
| C | -3.48396 | 1.219634 | -1.08068 |
| S | -2.74222 | 2.912673 | 0.703047 |
| C | -4.49485 | 2.179238 | -1.04775 |
| H | -3.4815 | 0.363801 | -1.74611 |
| C | -4.25982 | 3.189356 | -0.12816 |
| H | -5.38028 | 2.174661 | -1.66894 |
| C | 2.454042 | 1.474633 | 0.19726 |
| C | 3.484004 | 1.219717 | 1.080645 |
| S | 2.74233 | 2.912631 | -0.70322 |
| C | 4.494883 | 2.179332 | 1.0477 |
| H | 3.481529 | 0.363932 | 1.74614 |
| C | 4.259884 | 3.18939 | 0.128042 |
| C | 3.484002 | -1.2197 | -1.08069 |
| S | 2.742327 | -2.91261 | 0.703181 |
| C | 5.000552 | 4.34436 | -0.22352 |
| C | 5.000549 | -4.34434 | 0.223487 |
| C | -5.00049 | -4.34432 | -0.22341 |
| C | -5.00049 | 4.344332 | 0.223375 |
| H | 4.560402 | -4.95885 | 1.01376 |
| H | -4.56032 | -4.95888 | -1.01363 |
| H | -4.56032 | 4.958896 | 1.013591 |
| H | 4.560411 | 4.958871 | -1.0138 |
| C | 6.170271 | -4.86214 | -0.24396 |
| C | 7.092772 | -4.40719 | -1.30233 |
| C | 6.667242 | -6.13339 | 0.340571 |
| C | 8.158261 | -5.44477 | -1.35591 |
| C | -6.17025 | 4.862088 | -0.24404 |
| C | -7.0928 | 4.407061 | -1.30234 |
| C | -6.66721 | 6.133359 | 0.340444 |
| C | -7.91181 | 6.448989 | -0.40151 |
| C | -8.15832 | 5.444615 | -1.3559 |
| C | -9.28529 | 5.671387 | -2.08549 |
| C | -8.849 | 7.435235 | -0.408 |
| C | 7.911786 | -6.44909 | -0.40145 |
| C | 8.848956 | -7.43536 | -0.40794 |
| C | 9.28518 | -5.6716 | -2.08555 |
| C | 6.170271 | 4.862163 | 0.243933 |
| C | 7.092756 | 4.407209 | 1.302316 |
| S | 10.05971 | -7.13802 | -1.6008 |
| S | -10.0598 | 7.137809 | -1.60076 |
| O | 7.042458 | 3.421872 | 2.005905 |
| O | 7.042484 | -3.42185 | -2.00591 |
| O | 6.166486 | -6.78228 | 1.229176 |
| O | -6.16642 | 6.782316 | 1.228983 |
| O | -7.04253 | 3.421688 | -2.00588 |
| C | 7.911792 | 6.449102 | 0.401431 |
| C | 8.848965 | 7.435364 | 0.407927 |
| C | 9.285169 | 5.671619 | 2.08555 |
| C | 6.667245 | 6.13341 | -0.34058 |
| C | 8.158255 | 5.444783 | 1.355897 |
| S | 10.05972 | 7.13803 | 1.600798 |
| O | 6.166506 | 6.782304 | -1.2292 |
| C | -6.17024 | -4.86208 | 0.244007 |
| C | -7.09278 | -4.40704 | 1.302305 |
| C | -6.6672 | -6.13334 | -0.34047 |
| C | -8.15828 | -5.44462 | 1.355915 |
| C | -7.91177 | -6.449 | 0.401523 |
| C | -8.84893 | -7.43528 | 0.408056 |
| C | -9.28522 | -5.67142 | 2.085539 |
| O | -7.04251 | -3.42167 | 2.005847 |
| O | -6.1664 | -6.78231 | -1.229 |
| S | -10.0597 | -7.13787 | 1.600862 |
| H | 3.481526 | -0.36392 | -1.74619 |
| H | 5.380283 | 2.174807 | 1.668934 |
| H | 5.380282 | -2.1748 | -1.66898 |
| C | 8.86847 | -8.64424 | 0.465044 |
| C | 9.836455 | -4.81797 | -3.17774 |
| C | 9.836433 | 4.817991 | 3.177757 |
| C | 8.868485 | 8.644244 | -0.46506 |
| C | -8.86848 | 8.644166 | 0.464923 |
| C | -9.83661 | 4.817687 | -3.17761 |
| C | -8.86841 | -8.64422 | -0.46484 |
| C | -9.83653 | -4.81771 | 3.177661 |
| F | 8.972277 | -4.70158 | -4.18367 |
| F | 10.11104 | -3.59033 | -2.74337 |
| F | 10.96488 | -5.33622 | -3.66963 |
| F | 8.920929 | -8.31021 | 1.752601 |
| F | 7.772263 | -9.37956 | 0.290138 |
| F | 9.924456 | -9.41637 | 0.199763 |
| F | 8.97225 | 4.701621 | 4.183681 |
| F | 10.11102 | 3.59035 | 2.743406 |
| F | 10.96485 | 5.336247 | 3.669638 |
| F | 9.924479 | 9.416361 | -0.19979 |
| F | 7.772284 | 9.379569 | -0.29015 |
| F | -8.92082 | 8.3102 | 1.7525 |
| F | -7.77231 | 9.379504 | 0.289886 |
| F | -9.92451 | 9.416244 | 0.199689 |
| F | -8.9725 | 4.701277 | -4.18359 |
| F | -10.1111 | 3.590063 | -2.74317 |
| F | -10.9651 | 5.33589 | -3.66944 |
| F | -8.97238 | -4.70126 | 4.183609 |
| F | -10.1111 | -3.59011 | 2.743209 |
| F | -10.965 | -5.33594 | 3.669537 |
| F | -9.9244 | -9.41633 | -0.19956 |
| F | -7.77221 | -9.37952 | -0.28984 |
| F | 8.920935 | 8.310205 | -1.75262 |
| F | -8.92081 | -8.31028 | -1.75243 |

**Table S7:** Cartesian Coordinates of **TTB5** compound.

| **Atom** | **X-axis** | **Y-axis** | **Z-axis** |
| --- | --- | --- | --- |
| C | 1.218959 | -0.70447 | -0.04066 |
| C | -0.00012 | -1.37464 | -0.00032 |
| C | -1.21918 | -0.70445 | 0.040036 |
| C | -1.21919 | 0.70409 | -0.04053 |
| C | -0.0001 | 1.37425 | -0.00026 |
| C | 1.218969 | 0.704063 | 0.039962 |
| H | -0.00013 | -2.46135 | -0.00033 |
| H | -9.7E-05 | 2.460956 | -0.00024 |
| C | -2.45431 | 1.465168 | -0.22809 |
| C | -3.47236 | 1.177025 | -1.11669 |
| S | -2.77156 | 2.91368 | 0.642243 |
| C | -4.50237 | 2.113362 | -1.10428 |
| C | -4.29516 | 3.143773 | -0.19764 |
| H | -5.38379 | 2.077874 | -1.72888 |
| C | 2.454119 | 1.465091 | 0.22762 |
| C | 4.295019 | 3.143621 | 0.197431 |
| C | 4.502052 | 2.113233 | 1.104132 |
| C | -2.45433 | -1.4655 | 0.227727 |
| C | -3.47221 | -1.17734 | 1.116494 |
| S | -2.77173 | -2.91401 | -0.64255 |
| C | -4.50225 | -2.11366 | 1.104264 |
| C | -4.2952 | -3.14407 | 0.197587 |
| C | 2.454087 | -1.46553 | -0.22836 |
| C | 3.471949 | -1.17739 | -1.11716 |
| S | 2.771551 | -2.91393 | 0.64205 |
| C | 4.502015 | -2.11368 | -1.10487 |
| C | 4.29504 | -3.144 | -0.19807 |
| C | 3.472014 | 1.17691 | 1.116372 |
| S | 2.771545 | 2.913556 | -0.64271 |
| C | 5.062332 | -4.28095 | 0.144813 |
| C | 5.06226 | 4.280636 | -0.14535 |
| C | -5.06221 | 4.28092 | 0.145115 |
| C | -5.06242 | -4.28112 | -0.14513 |
| H | 4.612756 | 4.935435 | -0.89663 |
| H | -4.61249 | 4.935783 | 0.896205 |
| H | -4.61287 | -4.93597 | -0.89633 |
| H | 4.612829 | -4.93573 | 0.896109 |
| C | 6.278148 | 4.738448 | 0.273257 |
| C | 7.234079 | 4.186965 | 1.240386 |
| C | 6.796253 | 5.990385 | -0.31701 |
| C | 8.396573 | 5.144431 | 1.224654 |
| C | -6.27829 | -4.73895 | 0.273498 |
| C | -7.23431 | -4.18741 | 1.240508 |
| C | -6.79637 | -5.99091 | -0.31676 |
| C | -8.13709 | -6.20274 | 0.322828 |
| C | -8.39681 | -5.14486 | 1.224719 |
| C | -9.62058 | -5.28796 | 1.820805 |
| C | -9.17316 | -7.09403 | 0.28104 |
| C | 8.136863 | 6.202327 | 0.32277 |
| C | 9.172834 | 7.093728 | 0.281167 |
| C | 9.620229 | 5.287664 | 1.820938 |
| C | 6.278278 | -4.7387 | -0.27369 |
| C | 7.234275 | -4.18716 | -1.24072 |
| S | 10.49005 | 6.686796 | 1.313166 |
| S | -10.4905 | -6.68692 | 1.312773 |
| O | 7.135378 | -3.18925 | -1.91301 |
| O | 7.135353 | 3.188813 | 1.91235 |
| O | 6.249067 | 6.678054 | -1.14126 |
| O | -6.24914 | -6.67862 | -1.14096 |
| O | -7.13562 | -3.18926 | 1.912476 |
| C | 8.137278 | -6.20224 | -0.3227 |
| C | 9.173486 | -7.09335 | -0.28068 |
| C | 9.620854 | -5.28733 | -1.82051 |
| C | 6.796432 | -5.99057 | 0.316678 |
| C | 8.396987 | -5.14436 | -1.22459 |
| S | 10.49089 | -6.68619 | -1.31235 |
| O | 6.249181 | -6.6783 | 1.140844 |
| C | -6.27812 | 4.738803 | -0.27334 |
| C | -7.23434 | 4.187218 | -1.24012 |
| C | -6.796 | 5.990881 | 0.31684 |
| C | -8.39669 | 5.144847 | -1.22438 |
| C | -8.13675 | 6.202809 | -0.32265 |
| C | -9.17264 | 7.094297 | -0.28096 |
| C | -9.62044 | 5.288098 | -1.82047 |
| O | -7.1359 | 3.188896 | -1.91188 |
| O | -6.24854 | 6.678686 | 1.140805 |
| S | -10.4901 | 6.68732 | -1.31264 |
| O | 11.37918 | -4.7923 | -3.16283 |
| O | 9.682236 | -3.45774 | -3.1789 |
| O | 8.352761 | 8.64512 | -1.16914 |
| O | 10.35815 | 8.899332 | -0.40863 |
| O | 11.37823 | 4.793044 | 3.163836 |
| O | 9.681713 | 3.457936 | 3.179146 |
| O | 10.35898 | -8.89871 | 0.409445 |
| O | 8.353236 | -8.64505 | 1.169203 |
| O | -9.68188 | 3.458734 | -3.17917 |
| O | -11.3792 | 4.792862 | -3.16222 |
| O | -8.35317 | -8.6453 | -1.16946 |
| O | -10.3587 | -8.89923 | -0.40932 |
| O | -9.68163 | -3.45884 | 3.17984 |
| O | -8.35235 | 8.645639 | 1.16928 |
| O | -11.3791 | -4.79278 | 3.162886 |
| O | -10.3577 | 8.90009 | 0.408763 |
| N | 9.298783 | -8.30853 | 0.499697 |
| N | 10.27277 | -4.43844 | -2.7994 |
| N | 9.298038 | 8.309023 | -0.49905 |
| N | 10.27208 | 4.43886 | 2.799957 |
| N | -9.29838 | -8.30929 | -0.49922 |
| N | -10.2724 | -4.43917 | 2.799824 |
| N | -10.2725 | 4.439229 | -2.79929 |
| N | -9.29769 | 8.309607 | 0.499244 |
| H | 3.447029 | -0.31262 | -1.77027 |
| H | 5.383317 | -2.07822 | -1.72963 |
| H | 3.447129 | 0.312112 | 1.769444 |
| H | 5.383343 | 2.077785 | 1.728909 |
| H | -3.44762 | 0.312204 | -1.76975 |
| H | -3.44732 | -0.31253 | 1.76955 |
| H | -5.38355 | -2.07818 | 1.729028 |

**Table S8:** Cartesian Coordinates of **TTB6** compound.

| **Atom** | **X-axis** | **Y-axis** | **Z-axis** |
| --- | --- | --- | --- |
| C | -1.21966 | 0.704288 | -0.03571 |
| C | 1.219605 | 0.704287 | 0.035699 |
| C | 1.219603 | -0.70429 | -0.03573 |
| C | -2.7E-05 | -1.37386 | -1.8E-05 |
| C | -1.21966 | -0.70428 | 0.035699 |
| H | -2.5E-05 | 2.460517 | 1.1E-05 |
| H | -2.8E-05 | -2.46051 | -2.9E-05 |
| C | 2.455295 | -1.46959 | -0.20379 |
| C | 3.48152 | -1.20241 | -1.08897 |
| S | 2.761424 | -2.90103 | 0.699729 |
| C | 4.508467 | -2.1425 | -1.04841 |
| H | 3.465836 | -0.34968 | -1.75809 |
| C | 4.289004 | -3.15214 | -0.12275 |
| H | 5.396566 | -2.12321 | -1.66577 |
| C | -2.45536 | -1.46959 | 0.203735 |
| C | -4.28907 | -3.15213 | 0.122654 |
| C | -4.50855 | -2.14247 | 1.048302 |
| C | 2.4553 | 1.469593 | 0.20375 |
| C | 3.481535 | 1.202403 | 1.088913 |
| S | 2.761421 | 2.901032 | -0.69977 |
| C | 4.508482 | 2.142489 | 1.048346 |
| H | 3.465859 | 0.349667 | 1.758031 |
| C | 4.289009 | 3.15214 | 0.122691 |
| H | 5.396588 | 2.123205 | 1.665698 |
| C | -2.45535 | 1.469592 | -0.20373 |
| C | -3.48161 | 1.202395 | -1.08887 |
| S | -2.76147 | 2.901024 | 0.699804 |
| C | -4.50856 | 2.142478 | -1.04828 |
| H | -3.46594 | 0.349657 | -1.75799 |
| C | -4.28907 | 3.152132 | -0.12263 |
| C | -3.4816 | -1.20239 | 1.08888 |
| S | -2.76148 | -2.90102 | -0.6998 |
| C | -5.05773 | 4.282079 | 0.242793 |
| C | -5.05773 | -4.28207 | -0.24277 |
| C | 5.057674 | -4.28209 | 0.242663 |
| C | 5.057678 | 4.282086 | -0.24272 |
| H | -4.61373 | -4.92492 | -1.00765 |
| H | 4.613691 | -4.92493 | 1.007562 |
| H | 4.613693 | 4.924932 | -1.00762 |
| H | -4.61373 | 4.924924 | 1.007677 |
| C | -6.27245 | -4.7365 | 0.178505 |
| C | -7.22843 | -4.20663 | 1.167465 |
| C | -6.81492 | -5.97992 | -0.42348 |
| C | -8.36989 | -5.15934 | 1.14425 |
| C | 6.272392 | 4.736511 | 0.178574 |
| C | 7.228337 | 4.20664 | 1.167566 |
| C | 6.814876 | 5.979932 | -0.42339 |
| C | 8.136101 | 6.187664 | 0.22356 |
| C | 8.369878 | 5.159244 | 1.144258 |
| C | 9.569633 | 5.269271 | 1.791346 |
| C | 9.156378 | 7.096918 | 0.155951 |
| C | -8.13608 | -6.18776 | 0.223559 |
| C | -9.15628 | -7.09711 | 0.156042 |
| C | -9.56959 | -5.26946 | 1.79142 |
| C | -6.27246 | 4.736504 | -0.17847 |
| C | -7.22844 | 4.206626 | -1.16742 |
| S | -10.437 | -6.67598 | 1.253609 |
| S | 10.43712 | 6.675708 | 1.253457 |
| O | -7.16524 | 3.224067 | -1.87359 |
| O | -7.16523 | -3.22408 | 1.873631 |
| O | -6.30209 | -6.68215 | -1.26215 |
| O | 6.302092 | 6.682132 | -1.26212 |
| O | 7.165133 | 3.224054 | 1.873693 |
| C | -8.13609 | 6.187754 | -0.22352 |
| C | -9.15629 | 7.097102 | -0.156 |
| C | -9.5696 | 5.269446 | -1.79137 |
| C | -6.81493 | 5.979916 | 0.42352 |
| C | -8.3699 | 5.15933 | -1.14421 |
| S | -10.437 | 6.675967 | -1.25357 |
| O | -6.30209 | 6.682155 | 1.262183 |
| C | 6.272385 | -4.73652 | -0.17864 |
| C | 7.228329 | -4.20666 | -1.16764 |
| C | 6.814861 | -5.97994 | 0.423327 |
| C | 8.369884 | -5.15925 | -1.14429 |
| C | 8.136103 | -6.18766 | -0.22359 |
| C | 9.156392 | -7.0969 | -0.15595 |
| C | 9.569654 | -5.26927 | -1.79136 |
| O | 7.165136 | -3.22408 | -1.87376 |
| O | 6.30208 | -6.68213 | 1.262072 |
| S | 10.43715 | -6.67569 | -1.25344 |
| H | -3.46593 | -0.34965 | 1.757994 |
| H | -5.39668 | 2.12319 | -1.66562 |
| H | -5.39667 | -2.12318 | 1.665642 |
| C | -9.24145 | -8.24477 | -0.66042 |
| N | -9.31946 | -9.18512 | -1.32472 |
| C | -10.1023 | -4.40566 | 2.771459 |
| N | -10.5459 | -3.70532 | 3.574247 |
| C | -10.1023 | 4.405642 | -2.77141 |
| N | -10.5459 | 3.705302 | -3.57419 |
| C | -9.24145 | 8.244773 | 0.66045 |
| N | -9.31946 | 9.18512 | 1.324744 |
| C | 9.241596 | 8.244558 | -0.66054 |
| N | 9.319641 | 9.184878 | -1.32487 |
| C | 10.10235 | 4.405445 | 2.77137 |
| N | 10.54594 | 3.705086 | 3.574143 |
| C | 9.241608 | -8.24453 | 0.660558 |
| N | 9.319653 | -9.18484 | 1.324898 |
| C | 10.10238 | -4.40545 | -2.77138 |
| N | 10.54597 | -3.70509 | -3.57415 |

**Table S9**: The frontier orbital energies and their band gaps (*eV*) for the studied chromophores (**TTBR** and **TTB1-TTB6**).

| **Compounds** | ***E*_HOMO_** | ***E*_LUMO_** | **Δ*E*** |
| --- | --- | --- | --- |
| **TTBR** | -6.328 | -3.251 | 3.077 |
| **TTB1** | -6.419 | -3.255 | 3.164 |
| **TTB2** | -6.497 | -3.313 | 3.184 |
| **TTB3** | -6.503 | -3.357 | 3.146 |
| **TTB4** | -6.554 | -3.596 | 2.958 |
| **TTB5** | -6.658 | -4.118 | 2.540 |
| **TTB6** | -6.640 | -3.825 | 2.821 |

Band gap = *E*_LUMO_−*E*_HOMO_, units in *eV*

**Table S10**: Calculated energies (*E*) and energy gap (∆*E*) of HOMO-1, LUMO+1, HOMO-2 and LUMO+2 for **TTBR-TTB6.**

| **Compounds** | **HOMO-1** | **LUMO+1** | **∆*E*** | **HOMO-2** | **LUMO+2** | **∆*E*** |
| --- | --- | --- | --- | --- | --- | --- |
| **TTBR** | -6.695 | -2.960 | 3.735 | -6.792 | -2.913 | 3.879 |
| **TTB1** | -6.771 | -3.002 | 3.769 | -6.857 | -2.949 | 3.908 |
| **TTB2** | -6.848 | -3.064 | 3.784 | -6.923 | -3.030 | 3.893 |
| **TTB3** | -6.851 | -3.122 | 3.729 | -6.929 | -3.087 | 3.842 |
| **TTB4** | -6.937 | -3.351 | 3.586 | -7.021 | -3.302 | 3.719 |
| **TTB5** | -7.036 | -4.036 | 3.000 | -7.130 | -4.013 | 3.17 |
| **TTB6** | -7.018 | -3.645 | 3.436 | -7.110 | -3.601 | 3.509 |

**Table S11:** Wavelength (*λ_max_*$\mathbf{)}$, excitation energy (*E*), oscillator strength (*f*_os_) and nature of molecular orbital contributions of compounds (**TTBR** and **TTB1-TTB6**) in dichloromethane phases.

| **Compounds** | **DFT**  ***λ_max_* (*nm*)** | ***E* (*eV*)** | ***f*_os_** | **MO contributions** |
| --- | --- | --- | --- | --- |
| **TTBR** | 506.451 | 2.448 | 1.311 | H→L (93%) |
| **TTB1** | 490.774 | 2.526 | 1.330 | H→L (92%) |
| **TTB2** | 486.365 | 2.549 | 1.432 | H→L (92%) |
| **TTB3** | 493.509 | 2.512 | 1.439 | H→L (91%) |
| **TTB4** | 526.584 | 2.355 | 1.402 | H→L (93%) |
| **TTB5** | 605.895 | 2.046 | 0.916 | H→L (90%) |
| **TTB6** | 553.205 | 2.241 | 1.238 | H→L (91%) |

**Table S12:** Wavelength, excitation energy and oscillator strength of investigated compound in solvent phase (dichloromethane).

| **Compounds** | **DFT**  ***λ_max_* (*nm*)** | | ***E (eV)*** | | ***f_os_*** | | **MO contributions** |  |  |  |  |
| --- | --- | --- | --- | --- | --- | --- | --- | --- | --- | --- | --- |
|  | 506.451 | | 2.448 | | 1.311 | | H→L (93%), H-1→L+1 (4%) | |  |  |  |
|  | 460.925 | | 2.690 | | 0.017 | | H-1→L (39%), H→L+1 (58%), | |  |  |  |
| **TTBR** | 445.842 | | 2.781 | | 2.799 | | H-2→L (14%), H→L+2 (78%), H-1→L+3 (5%) | |  |  |  |
|  | 424.429 | | 2.921 | | 0.550 | | H-2→L (80%), H→L+2 (16%), | |  |  |  |
|  | 419.716 | | 2.954 | | 0.001 | | H-1→L (58%), H→L+1 (40%), | |  |  |  |
|  | 406.692 | | 3.049 | | 0.000 | | H→L+3 (90%), H-3→L (3%), H-1→L+2 (3%) | |  |  |  |
|  | | 490.774 | | 2.526 | | 1.330 | H→L (92%), H-1→L+1 (4%) | | |  |  |
|  | | 451.837 | | 2.744 | | 0.069 | H-1→L (34%), H→L+1 (58%), H→L+2 (2%) | | |  |  |
| **TTB1** | | 439.131 | | 2.823 | | 2.429 | H-2→L (13%), H→L+2 (65%), H-1→L+3 (6%), H→L+1 (7%) | | |  |  |
|  | | 419.901 | | 2.953 | | 0.008 | H-3→L (14%), H-3→L+2 (12%), H-3→L+3 (16%), H-2→L (15%), H→L+2 (12%), H-4→L (4%), H-4→L+2 (4%), H-4→L+3 (5%), H-3→L+1 (4%) | | |  |  |
|  | | 414.012 | | 2.995 | | 0.621 | H-2→L (62%), H-4→L+1 (3%), H-3→L (9%), H-3→L+3 (3%), H→L+1 (6%), H→L+2 (5%) | | |  |  |
|  | | 410.286 | | 3.022 | | 0.010 | H-1→L (54%), H→L+1 (27%), H-3→L (3%), H→L+2 (9%) | | |  |  |
|  | | 486.365 | | 2.549 | | 1.432 | H→L (92%), H-1→L+1 (5%) | | | |  |
|  | | 448.795 | | 2.763 | | 0.015 | H-1→L (34%), H→L+1 (62%), | | | |  |
| **TTB2** | | 437.983 | | 2.831 | | 2.547 | H-2→L (13%), H→L+2 (78%), H-1→L+3 (6%) | | | |  |
|  | | 413.901 | | 2.996 | | 0.517 | H-2→L (80%), H→L+2 (16%), H-3→L+1 (2%) | | | |  |
|  | | 408.031 | | 3.039 | | 0.000 | H-1→L (61%), H→L+1 (36%), | | | |  |
|  | | 402.298 | | 3.082 | | 0.000 | H→L+3 (91%), H-3→L (2%), H-1→L+2 (3%) | | | |  |
|  | | 493.509 | | 2.512 | | 1.439 | H→L (91%), H-1→L+1 (5%) | | | |  |
|  | | 456.798 | | 2.714 | | 0.012 | H-1→L (33%), H→L+1 (62%), H-2→L+3 (2%) | | | |  |
| **TTB3** | | 444.946 | | 2.787 | | 2.709 | H-2→L (13%), H→L+2 (77%), H-1→L+3 (6%) | | | |  |
|  | | 418.173 | | 2.965 | | 0.548 | H-2→L (79%), H→L+2 (16%), | | | |  |
|  | | 412.909 | | 3.003 | | 0.000 | H-1→L (62%), H→L+1 (35%), | | | |  |
|  | | 408.690 | | 3.034 | | 0.000 | H→L+3 (91%), H-1→L+2 (3%) | | | |  |
|  | | 526.584 | | 2.355 | | 1.402 | H→L (93%), H-1→L+1 (4%) | | | |  |
|  | | 481.791 | | 2.573 | | 0.032 | H-1→L (29%), H→L+1 (67%), | | | |  |
| **TTB4** | | 467.107 | | 2.654 | | 2.387 | H-2→L (10%), H→L+2 (82%), H-1→L+3 (5%) | | | |  |
|  | | 439.458 | | 2.821 | | 0.681 | H-2→L (84%), H→L+2 (12%), | | | |  |
|  | | 435.582 | | 2.846 | | 0.004 | H-1→L (67%), H→L+1 (31%), | | | |  |
|  | | 427.723 | | 2.899 | | 0.000 | H→L+3 (93%), H-1→L+2 (2%) | | | |  |
|  | | 605.895 | | 2.046 | | 0.916 | H→L (90%), H-1→L+1 (6%) | | | | |
|  | | 577.799 | | 2.146 | | 0.017 | H-1→L (13%), H→L+1 (83%), | | | | |
| **TTB5** | | 565.673 | | 2.192 | | 1.188 | H→L+2 (89%), H-2→L (3%), H-1→L+3 (6%) | | | | |
|  | | 540.589 | | 2.294 | | 0.000 | H→L+3 (94%), H-1→L+2 (4%) | | | | |
|  | | 498.549 | | 2.487 | | 0.007 | H-1→L (77%), H→L+1 (16%), H-2→L+3 (4%) | | | | |
|  | | 493.725 | | 2.511 | | 0.872 | H-2→L (80%), H-3→L+1 (4%), H-2→L+4 (2%), H-1→L+3 (7%), H→L+2 (6%) | | | | |
|  | | 553.205 | | 2.241 | | 1.238 | H→L (91%), H-1→L+1 (5%) | | | | |
|  | | 512.437 | | 2.420 | | 0.021 | H-1→L (23%), H→L+1 (73%), | | | | |
| **TTB6** | | 496.155 | | 2.499 | | 2.176 | H→L+2 (85%), H-2→L (6%), H-1→L+3 (5%) | | | | |
|  | | 460.087 | | 2.695 | | 0.000 | H→L+3 (95%), H-1→L+2 (3%) | | | | |
|  | | 457.034 | | 2.713 | | 0.904 | H-2→L (85%), H-3→L+1 (3%), H→L+2 (8%) | | | | |
|  | | 456.311 | | 2.717 | | 0.004 | H-1→L (72%), H→L+1 (25%) | | | | |

MO=molecular orbital, H=HOMO, L=LUMO, *f*_os_*=* oscillator strength, wavelength= $\lambda$ (*nm*)

**Table S13:** Percentages of Acceptor and π-spacer for LUMOs and HOMOs

| **Compounds** | **LUMO** | | **HOMO** | |
| --- | --- | --- | --- | --- |
|  | **Acceptor** | **𝜋-Spacer** | **Acceptor** | **𝜋-Spacer** |
| **TTBR** | 56.4 | 43.6 | 29.4 | 70.6 |
| **TTB1** | 58.1 | 41.9 | 29.2 | 70.8 |
| **TTB2** | 57.6 | 42.4 | 28.7 | 71.3 |
| **TTB3** | 60.8 | 39.2 | 29.4 | 70.6 |
| **TTB4** | 62.4 | 37.6 | 29.4 | 70.6 |
| **TTB5** | 86.0 | 14.0 | 30.6 | 69.4 |
| **TTB6** | 71.0 | 29.0 | 29.9 | 70.1 |


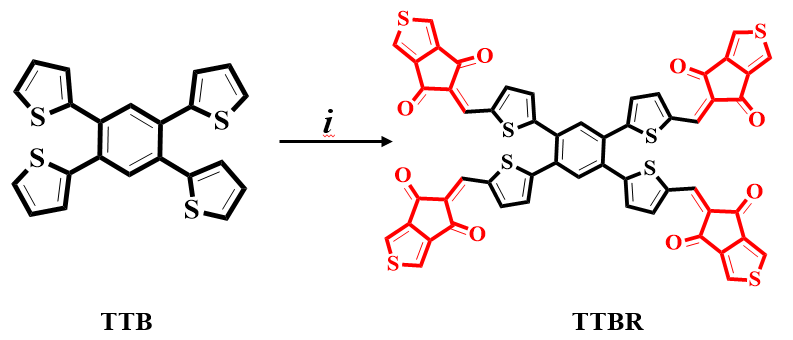
**Figure S1**: Modification of **TTB** into **TTBR**; (i) *via* different acceptor moieties.

|  |  |  |
| --- | --- | --- |
| **(A1)** | **(A2)** | **(A3)** |
|  |  |  |
| **(A4)** | **(A5)** | **(A6)** |
|  |  |  |
| **(A7)** |  |  |

**Figure S2**: The structures of various utilized acceptors in the structural tailoring of **TTBR**.

|  |  |
| --- | --- |
| **TTBR** | **TTB1** |
|  |  |
| **TTB2** | **TTB3** |
|  |  |
| **TTB4** | **TTB5** |
|  | |
| **TTB6** | |


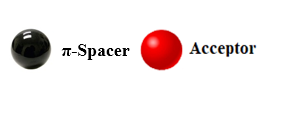


**Figure S3**: The structural view of designed chromophores with different acceptors

| **HOMO-1** | **LUMO+1** |
| --- | --- |
| 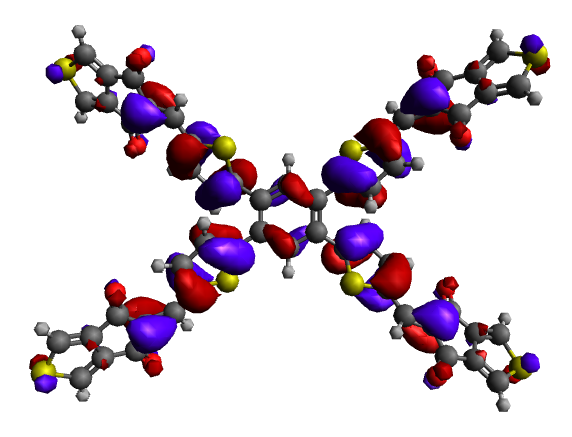 | 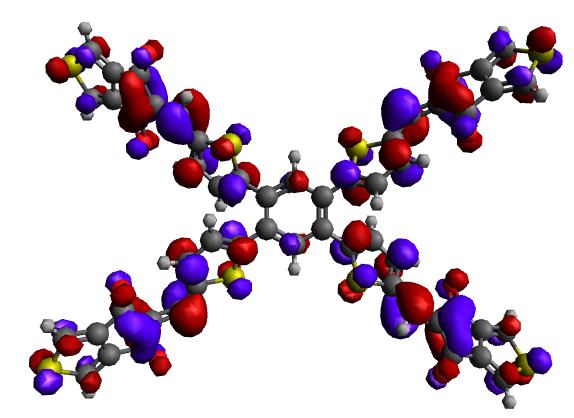 |
| **TTBR** | |
| 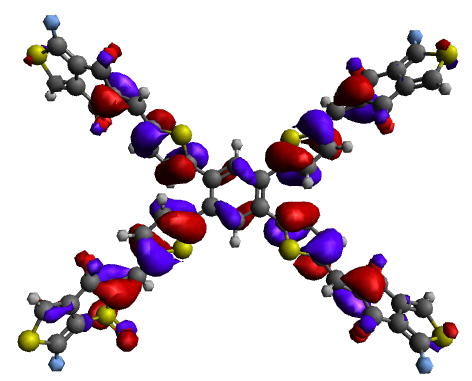 | 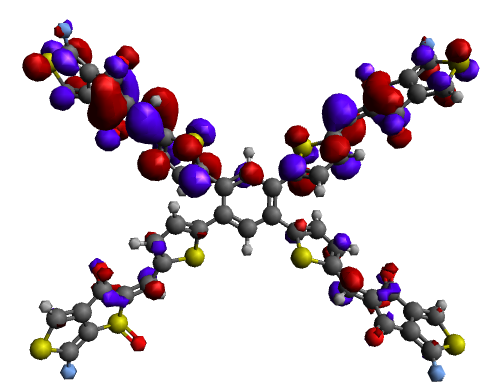 |
| **TTB1** | |
| 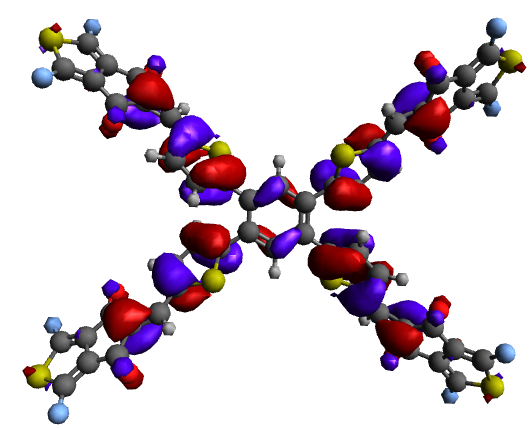 | 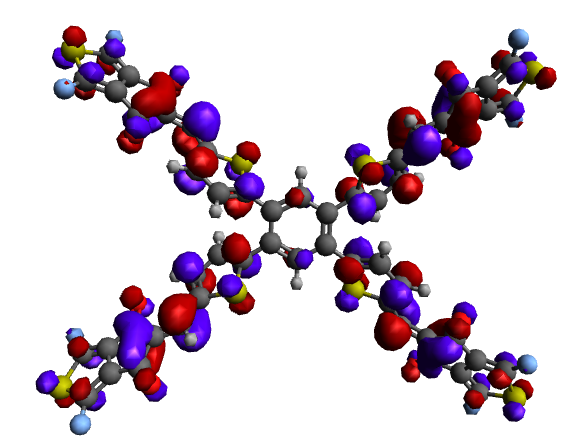 |
| **TTB2** | |
| 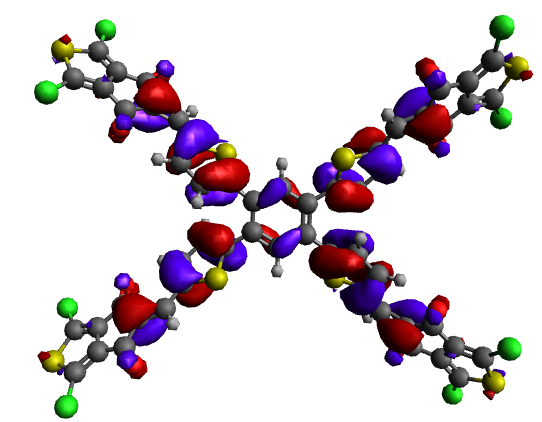 | 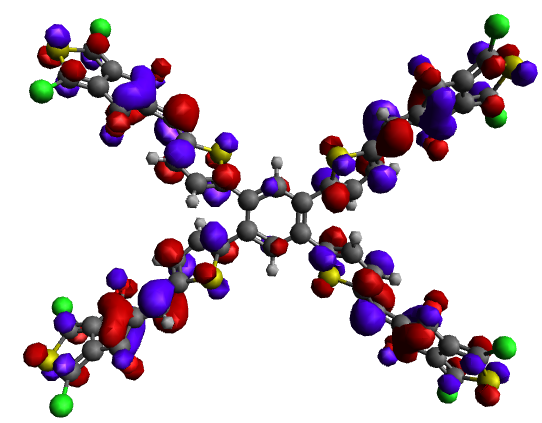 |
| **TTB3** | |
| 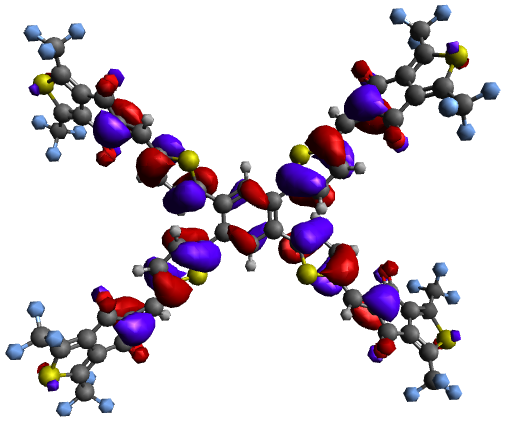 | 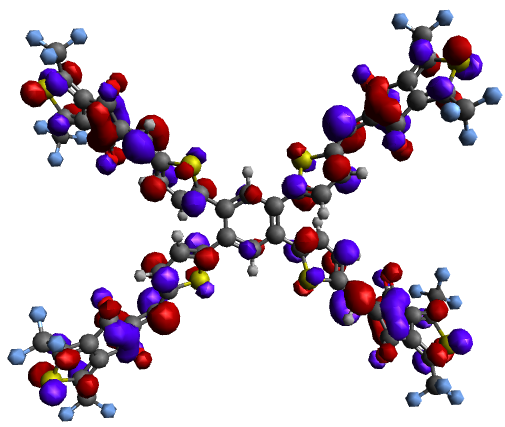 |
| **TTB4** | |
| 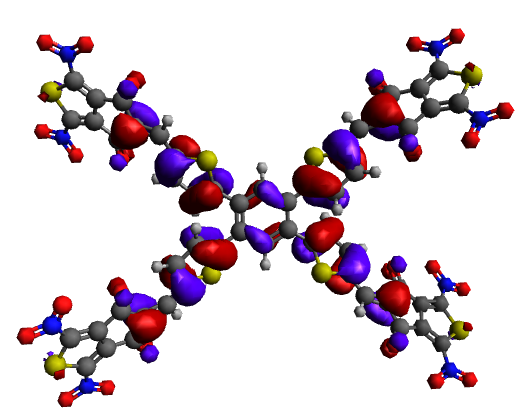 | 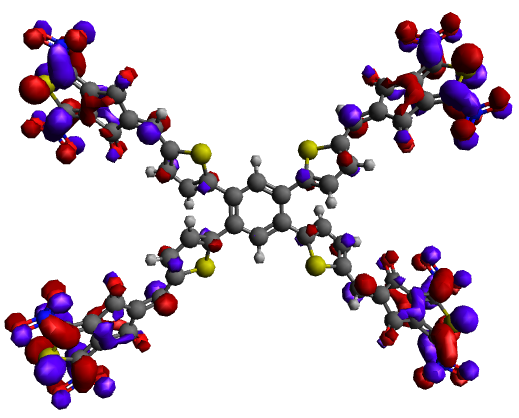 |
| **TTB5** | |
| 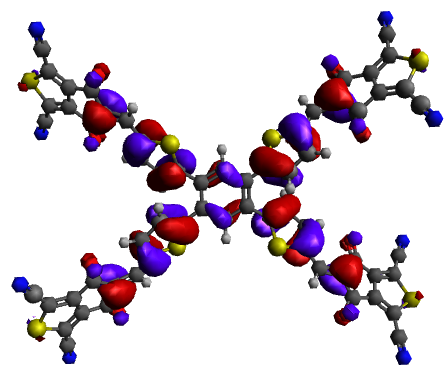 | 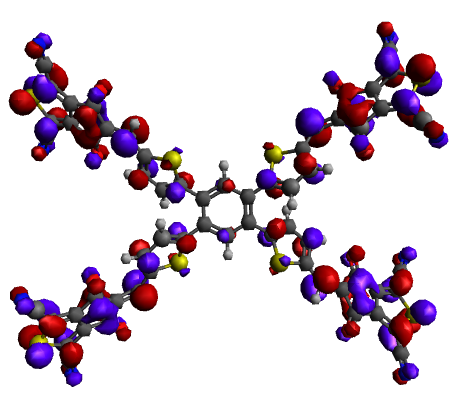 |
| **TTB6** | |
|  | |

| **HOMO-2** | **LUMO+2** |
| --- | --- |
| **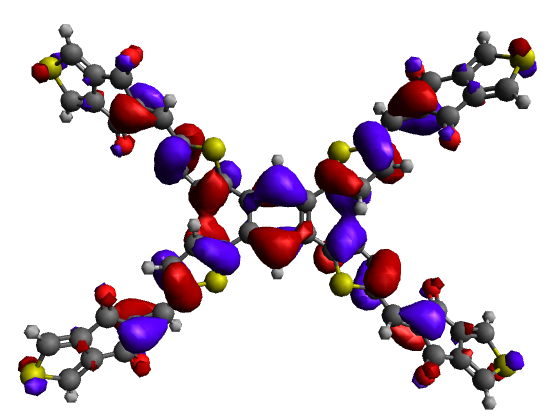** | **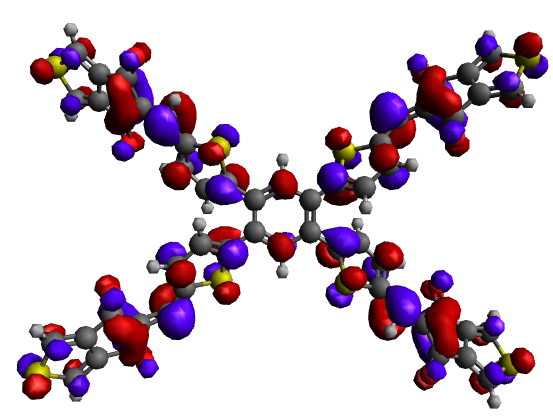** |
| **TTBR** | |
| **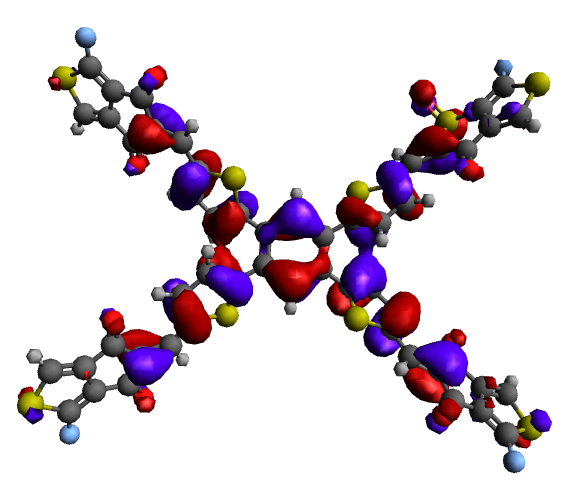** | **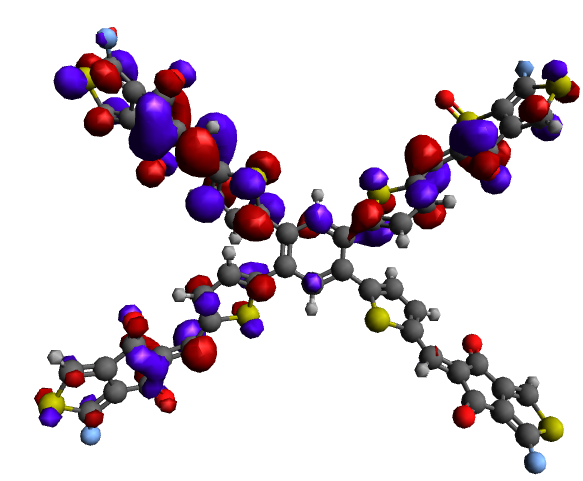** |
| **TBB1** | |
| **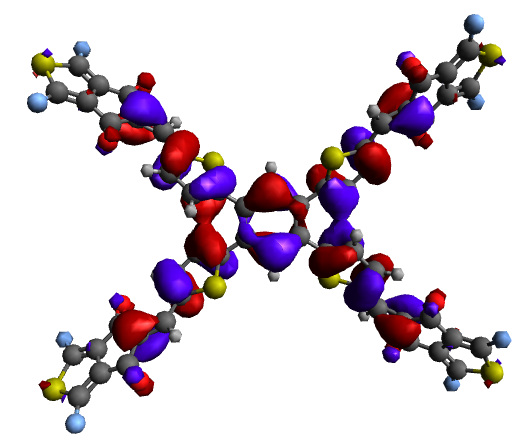** | **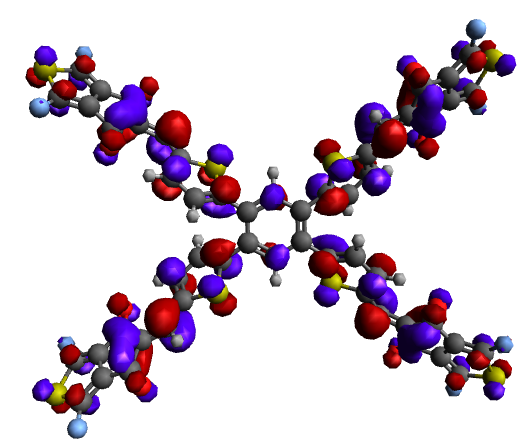** |
| **TBB2** | |
| **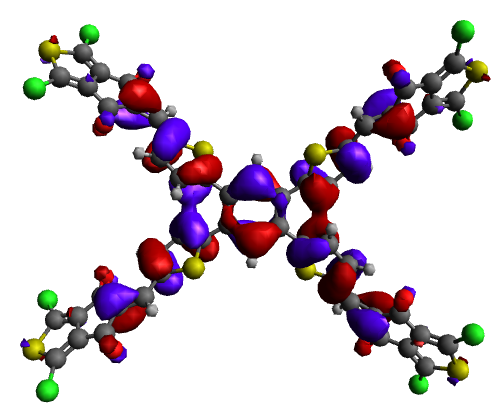** | **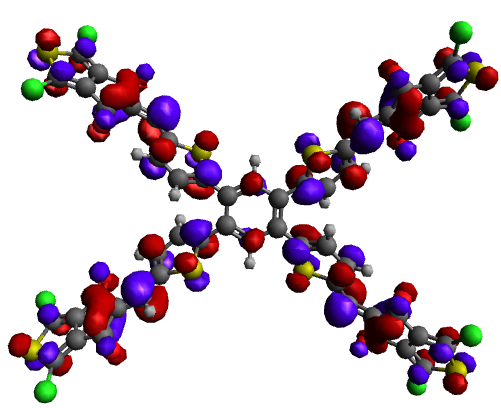** |
| **TBB3** | |
| **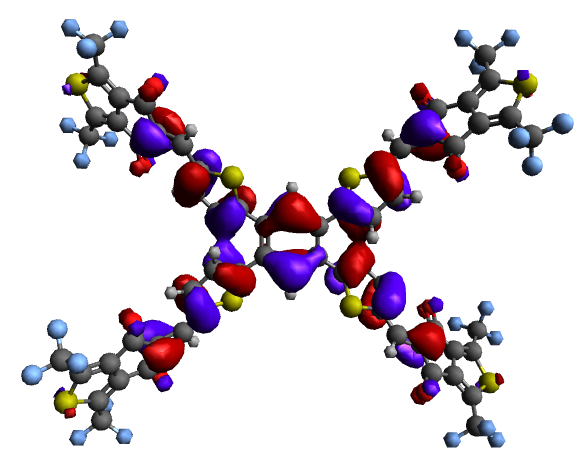** | **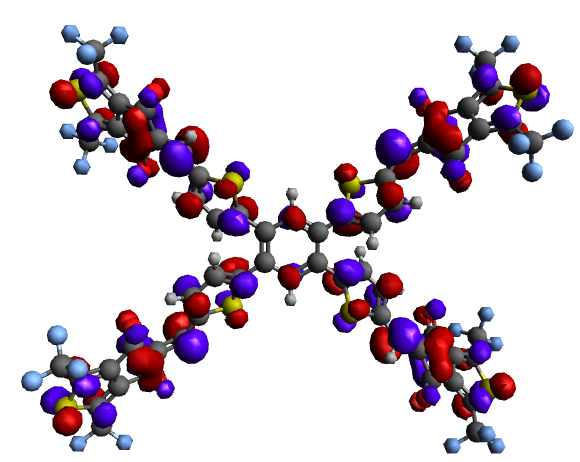** |
| **TBB4** | |
| **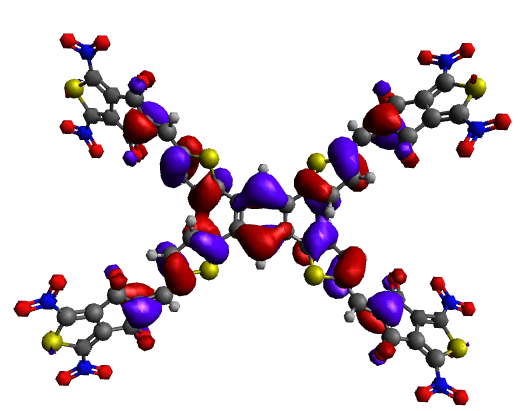** | **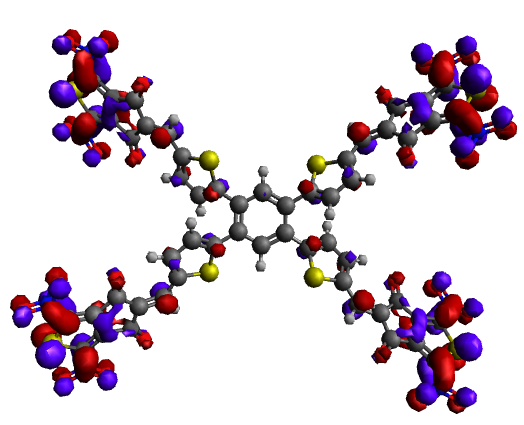** |
| **TBB5** | |
| **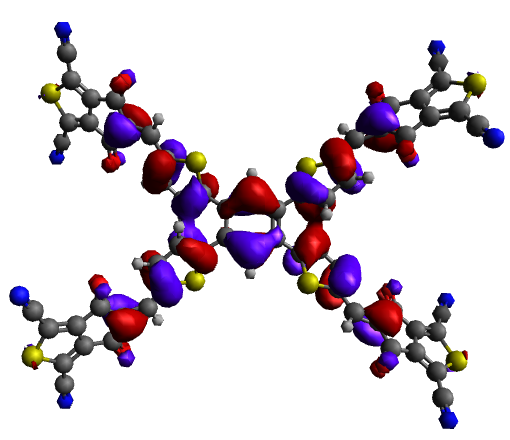** | **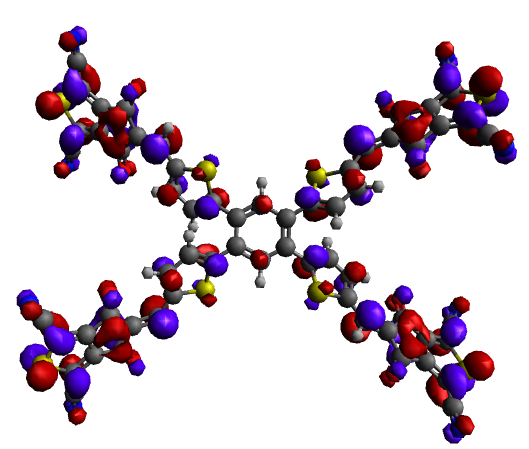** |
| **TBB6** | |

**Figure S4**: HOMO-1/LUMO+1 and HOMO-2/LUMO+2 of the analyzed compounds (**TTBR** and **TTB1-TTB6**).

| $X=\frac{\left[ IP+EA \right]}{2}$ | (S1) |
| --- | --- |
| $\eta=[IP-EA]$ | (S2) |
| $\mu=\frac{E_{HOMO}{+E}_{LUMO}}{2}$ | (S3) |
| $\sigma=\frac{1}{\eta}$ | (S4) |
| $\omega=\frac{\mu^{2}}{2\eta}$ | (S5) |
